# Targeting ERRα promotes cytotoxic effects against acute myeloid leukemia through suppressing mitochondrial oxidative phosphorylation

**DOI:** 10.1186/s13045-022-01372-7

**Published:** 2022-10-26

**Authors:** Wonhyoung Seo, Seungyeul Yoo, Yi Zhong, Sang-Hee Lee, Soo-Yeon Woo, Hee-Seon Choi, Minho Won, Taylor Roh, Sang Min Jeon, Kyeong Tae Kim, Prashanta Silwal, Min Joung Lee, Jun Young Heo, Nathan Lawlor, Sup Kim, Dongjun Lee, Jin-Man Kim, Ik-Chan Song, Jun Zhu, Eun-Kyeong Jo

**Affiliations:** 1 Department of Medical Science and Infection Control Convergence Research Center, Chungnam National University College of Medicine, Daejeon, South Korea; 2 Infection Control Convergence Research Center, Chungnam National University College of Medicine, Daejeon, South Korea; 3 Department of Biochemistry, Chungnam National University College of Medicine, Daejeon, South Korea; 4 Department of Pathology, Chungnam National University College of Medicine, Daejeon, South Korea; 5 Division of Hematology/Oncology, Department of Internal Medicine, Chungnam National University College of Medicine, Daejeon, South Korea; 6 Sema4, Stamford, CT USA; 7 Tisch Cancer Institute, Icahn School of Medicine at Mount Sinai, New York, NY USA; 8 Center for Research Equipment, Korea Basic Science Institute, Cheongju, South Korea; 9 Department of Convergence Medicine, School of Medicine, Pusan National University, Yangsan, South Korea; 10 Biotechnology Process Engineering Center, Korea Research Institute of Bioscience and Biotechnology, Cheongju, South Korea; 11 Department of Radiation Oncology, Chungnam National University Hospital, Daejeon, South Korea

**Keywords:** AML, ERRα, Mitochondrial oxidative phosphorylation, Apoptosis

## Abstract

Acute myeloid leukemia (AML) is an aggressive blood cancer with poor clinical outcomes. Emerging data suggest that mitochondrial oxidative phosphorylation (mtOXPHOS) plays a significant role in AML tumorigenesis, progression, and resistance to chemotherapies. However, how the mtOXPHOS is regulated in AML cells is not well understood. In this study, we investigated the oncogenic functions of ERRα in AML by combining in silico, in vitro, and in vivo analyses and showed ERRα is a key regulator of mtOXPHOS in AML cells. The increased ERRα level was associated with worse clinical outcomes of AML patients. Single cell RNA-Seq analysis of human primary AML cells indicated that ERRα-expressing cancer cells had significantly higher mtOXPHOS enrichment scores. Blockade of ERRα by pharmacologic inhibitor (XCT-790) or gene silencing suppressed mtOXPHOS and increased anti-leukemic effects in vitro and in xenograft mouse models.

**Supplementary Information:**

The online version contains supplementary material available at 10.1186/s13045-022-01372-7.

**To the Editor,**

Acute myeloid leukemia (AML) is the most common type of leukemia with an unsatisfactory clinical outcomes (5-year survival = 24%) [1, 2]. While recent studies have highlighted the significance of excessive mitochondrial respiration, metabolism, and oxidative phosphorylation (mtOXPHOS) in leukemogenesis [3–5], the key regulators of mitochondrial function in leukemic cells remain unknown. In this study, we report that, estrogen-related receptor-α (ERRα), an orphan nuclear receptor involved in mitochondrial biogenesis and metabolic homeostasis [6, 7], plays an oncogenic role in AML by combining in silico, in vitro, and in vivo analyses.

We first investigated whether ERRα expression is associated with AML tumorigenesis and progression. ERRα expression was significantly higher in leukemic cells than in hematopoietic stem and progenitor cells from healthy donors (Fig. 1A), in the bone marrow of AML patients than in healthy controls (Fig. 1B), and in AML cell lines than its level in normal immune cells (Fig. 1C). Immunohistochemistry staining further confirmed ERRα is expressed in bone marrow of AML patients but not of non-leukemia controls (Fig. 1D). Furthermore, ERRα expression was associated with patient survival rates in two independent AML cohorts (Fig. 1E). These results together suggest that ERRα plays an important role in AML tumorigenesis and progression. As a transcription factor binding promoter regions of its target genes [6–8], ERRα target genes in myeloid leukemia cells were identified by intersecting genes with predicted ERRα binding sites in their promoter regions, and genes co-expressed with ERRα in AML cell lines (Fig. 1F, Additional file 1: Data 1). ERRα activity scores based on the target genes were associated with patients’ survival (Additional file 3: Fig. S1A and B). The ERRα^+^ target genes were significantly enriched in the mtOXPHOS pathway (Fig. 1G, Additional file 3: Table S1) suggesting ERRα as a regulator of the mtOXPHOS pathway in AML cells.

**Fig. 1:**
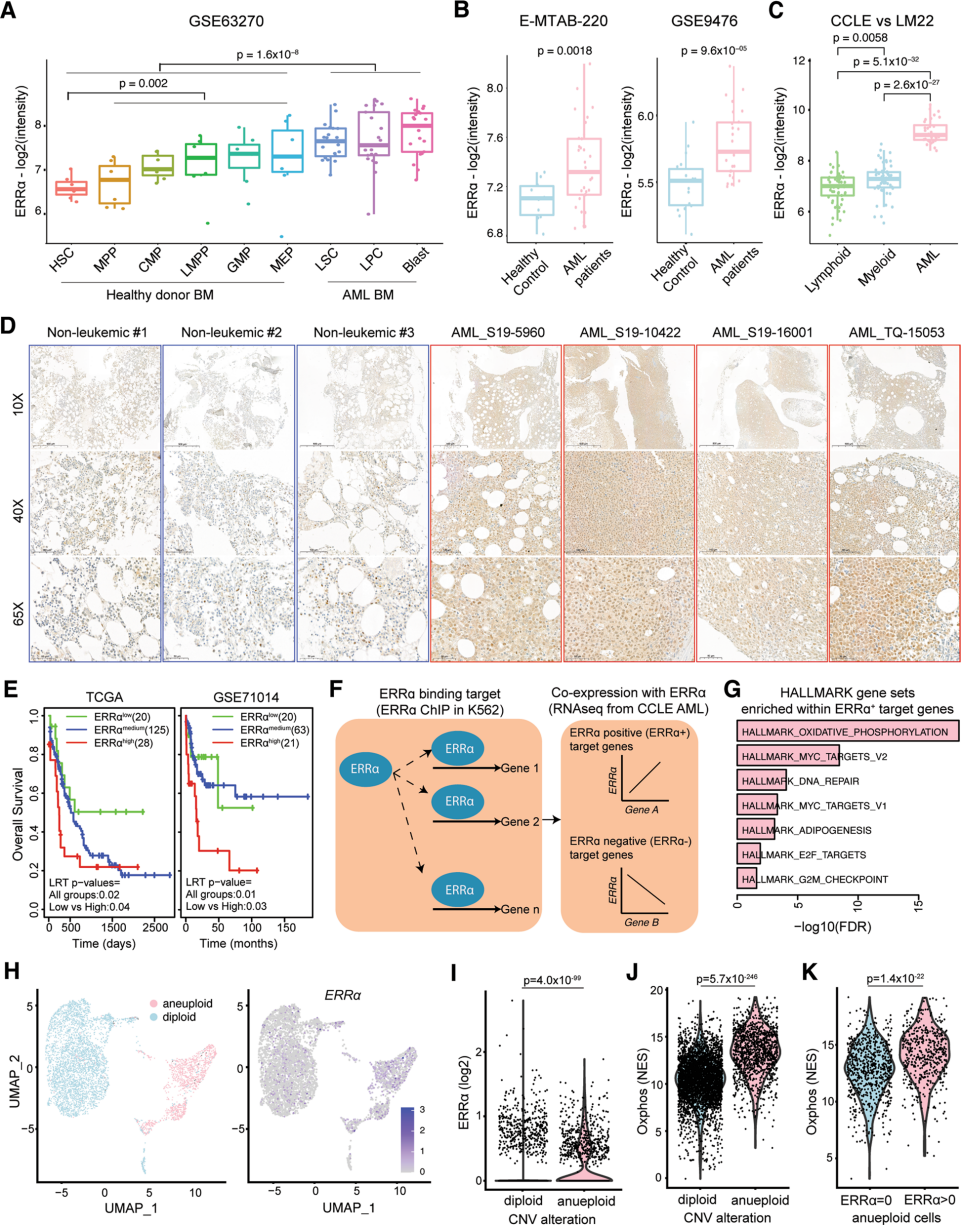
ERRα expression and OXPHOS pathway higher in AML cells.** A** ERRα expression comparison between hematopoietic stem or progenitor cells in 7 healthy donors and leukemic sub-population in 21 AML patients. Subtype information as well as CD34 status of individual sample is available in GSE63270; Hematopoietic stem cell (HSC), Multipotent progenitors (MPP), Common Myeloid Progenitor (CMP), Lymphoid-primed multipotent progenitor (LMPP), Granulocyte-erythroid progenitor (GMP), Megakaryocyte-erythroid progenitors (MEP). **B** ERRα expression comparison between healthy controls and AML patients in two independent cohorts (E-MTAB-220 and GSE9476). **C** ERRα expression comparison between AML cell lines (*n* = 32) and LM22 immune reference cells (*n* = 195). **A–C** *P* values were calculated by two-tailed *t* test. **D** Immunohistochemistric analysis of ERRα protein expression in bone marrow samples from three non-leukemic donors and four AML patients. **E** KM plots showing survival probability differences among patients stratified by mean and standard deviation of ERRα expression into low, medium, and high groups. *P* values were calculated by log-rank test (LRT). **F** Schematic definition of ERRα target genes from integration of ChiP and RNA-Seq data. **G** HALLMARK genes set significantly enriched (FDR < 0.01) within ERRα + genes in AML cell lines. **H** Clustering 5162 cells into aneuploid, and diploid cells based on copy number alterations determined by CopyKat (left). ERRα expression among the 5162 cells (right). **I** Comparison of ERRα expression between aneuploid and diploid cells. **J** Comparison of OXPHOS pathway activity between individual aneuploid and diploid cells. **K** Comparison of OXPHOS pathway activity between ERRα expressing aneuploid cells and other aneuploid cells. **(I–K)** *P* values were measured by two-tailed Wilcoxon rank sum test. Each dot in the figure represents a single cell

At the single-cell level, ERRα was expressed significantly higher in aneuploid compared to diploid cells (Fig. 1H and I). mtOXPHOS genes were expressed at significantly higher levels in aneuploid than diploid cells (Fig. 1J) and ERRα-expressing aneuploid cells showed significantly higher mtOXPHOS enrichment scores than aneuploid cells without ERRα expression (Fig. 1K). In the three AML samples from van Galen et al. [9], mtOXPHOS genes were expressed at higher levels in the ERRα-expressing malignant cells than in normal or other malignant cells (Additional file 3: Fig. S1C and D) confirming that ERRα expression is associated with higher mtOXPHOS in AML cells. From the transcriptomic profiling of KG1α cells with control and treatment of XCT-790 (an ERRα inverse agonist [10, 11]), the differentially expressed genes (Additional file 2: Data 2) significantly overlapped with the ERRα target genes, validating that transcription levels of the ERRα target genes were regulated by ERRα. XCT-790 treatment significantly downregulated the mtOXPHOS pathway and mitochondrial genes (Fig. 2A, Additional file 3: Table S2).

**Fig. 2:**
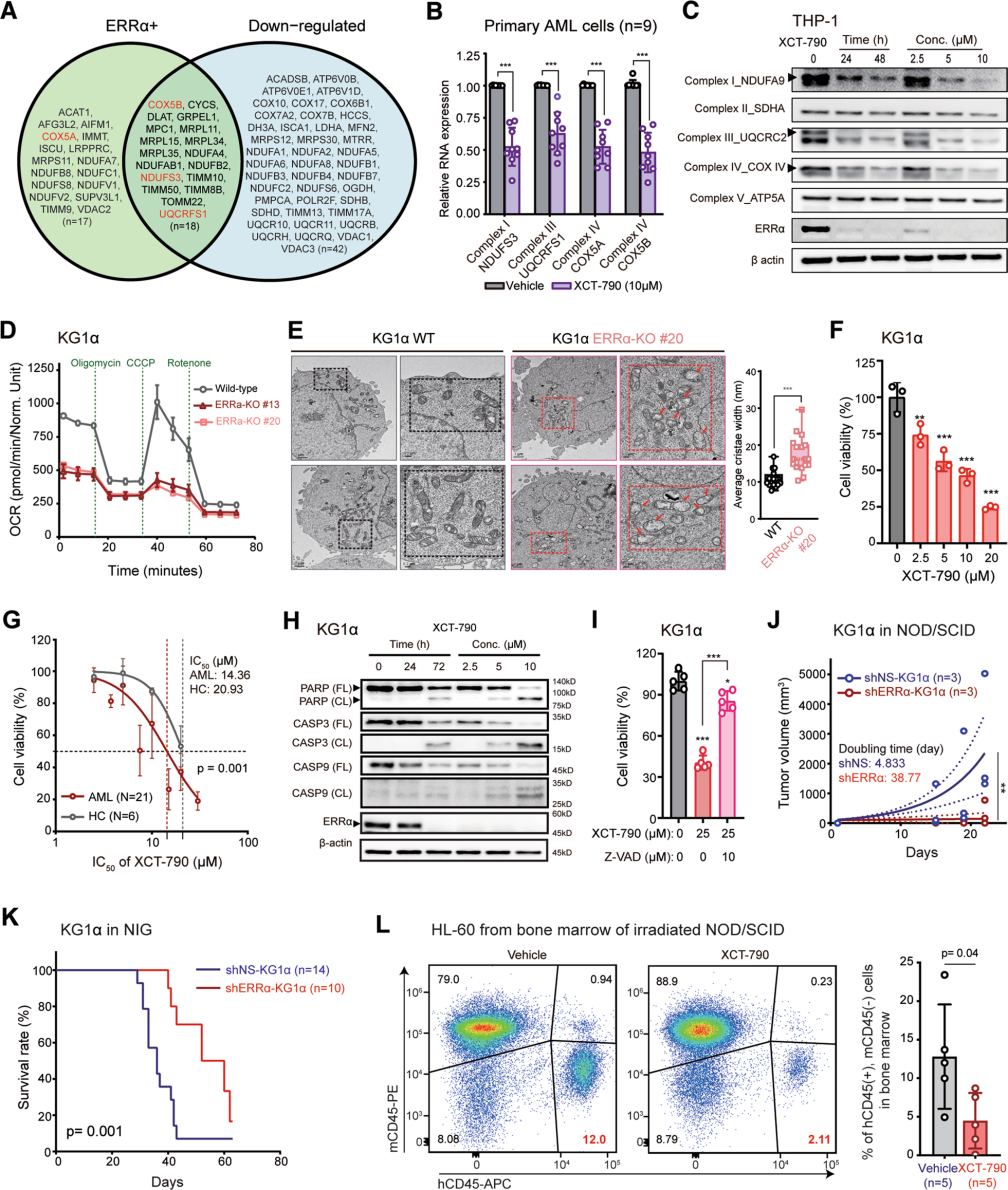
ERRα inhibition induces AML cell death through intrinsic apoptosis.** A** Venn diagram showing the overlap between down-regulated genes by XCT-790 treatment in RNA-Seq and ERRα + genes among HALLMARK OXPHOS genes. Four genes selected for further experimental validations were marked in red. **B** Relative expression of *NDUFS3, UQCRFS1, COX5A,* and *COX5B* significantly downregulated by XCT-790 treatment (10 µM for 24 h) in AML patient-derived cells (*n* = 9). **C.** Western analysis of mtOXPHOS complexes in THP-1 cells by XCT-790 treatment (5 µM; for lanes 2 and 3, 24 and 48 h, respectively) and at multiple concentrations (2.5 µM, 5 µM, 10 µM; 48 h). **D** Oxygen consumption rate (OCR) evaluated by Seahorse XF analysis between wild-type and ERRα knockout (KO #13 and #20) cells. **E** Representative electron microscopic images between wild-type (WT) and ERRα KO KG1α cells. Damaged, swollen, and disturbed cristae in the mitochondria of ERRα KO KG1α cells are marked with arrows. Scale bars, 1 µM and 0.2 µM. Quantification of the cristae width between WT (*n* = 20) and ERRα KO (*n* = 18) cells (right). **F**, **G**, and **I** CCK8 assay for KG1α cells (**F**, **I**), patient-derived AML cells, and primary monocytes from healthy controls (HC) (**G**). **F** and **G**, XCT-790 for 72 h; **I**, XCT-790 and/or Z-VAD-FMK (Z-VAD) for 30 h. **H** Western analysis of apoptotic proteins in KG1α cells by XCT-790 treatment (5 µM; for lanes 2 and 3, 24 and 72 h, respectively) and at multiple concentrations (2.5 µM, 5 µM, 10 µM; 72 h). **J** Progression of tumor volumes in NOD/SCID mice subcutaneously injected with KG1α cells transduced with targeting ERRα (shERRα-KG1α) or non-targeting control shRNA lentivirus (shNS-KG1α). **K**. Survival rates of NIG mice injected with shERRα-KG1α or shNS-KG1α (4 × 10^6^ cells/mice). Median survival times are 56 and 36 days for the shERRα-KG1α-engrafted mice (*n* = 10) and the shNS-KG1α-engrafted mice (*n* = 14), respectively. **L** Flow cytometric analysis of engrafted HL-60 cells into NOD/SCID mice at 4 weeks post-transplantation. A representative image of the engrafted HL-60 cells (human CD45+ (hCD45+) and murine CD45− (mCD45−)) by XCT-790 (8 mg/kg) for three weeks (left); the quantitative data of tumor burdens in the bone marrows (right). *P* < 0.05 (*), *P* < 0.01 (**) and *P* < 0.001 (***) were used to determine statistically significant differences. Two-tailed *t* test (**B**, **E** right, **L** right), extra sum of square F test (**G**, **J**), log-rank test (**K**) or one-way ANOVA (**F**, **I**). Data are the combined results from three independent experiments (**K**), representative of three independent experiments (**C**, **E** left, **H**, and **L** left). Data represent means ± SD from three or four independent experiments performed in triplicate (**B**, **D**, **E** right, **F**, **G**, **I**, **J**, and **L** right)

The associations between ERRα and the mtOXPHOS pathway were further investigated using 3 AML cell lines with high ERRα expression and mixed CD34 expression (Additional file 3: Fig. S2A) and primary cells (Additional file 3: Table S3). First, ERRα inhibition by either XCT-790 or shRNA specific to ERRα (shERRα) significantly reduced the mRNA expression of mtOXPHOS complexes (*NDUFS3*, *UQCRFS1*, *COX5A*, and *COX5B*) (Fig. 2B, and Additional file 3: Fig. S2B). In addition, ERRα blockade suppressed protein levels of mtOXPHOS complexes in AML cell lines (Fig. 2C and Additional file 3: Fig. S2C; Complex I, III, and IV by XCT-790 and Complex I and III by shERRα, respectively). Notably, XCT-790 treatment decreased the levels of mtOXPHOS complexes (Complex I, III, and IV in THP-1 cells) in the presence or absence of Z-VAD (Additional file 3: Fig. S2D), a pan-caspase inhibitor, suggesting that these proteins are suppressed by ERRα inhibition, not by cell death (Additional file 3: Fig. S2D). Further, cellular respiration and ATP generation were significantly decreased with ERRα targeting either by genetic knockout (Fig. 2D) or XCT-790 treatment (Additional file 3: Fig. S2E) in AML cell lines. Again, a decrease in basal/maximal respiration as well as a loss of ATP production was observed in XCT-790-treated cells independent from Z-VAD treatment (Additional file 3: Fig. S2E), indicating that the OCR differences were driven by ERRα inhibition rather than cell death. ERRα silencing also increased the number of damaged mitochondria with swollen and distorted cristae structures (Fig. 2E and Additional file 3: Fig. S2F), leading to decrease cell proliferation (Additional file 1: Fig. S2G). XCT-790 treatment decreased cell viability in AML cells (Fig. 2F and 2G, Additional file 3: Fig. S2H). More importantly, XCT-790 showed significantly stronger cytotoxicity to AML cells compared to normal monocytes (Fig. 2G), highlighting its potential as a therapeutic target. XCT-790 treatment in AML cells stimulated caspase 9 cleavage and apoptosis (Fig. 2H and I, Additional file 3: Fig S2I–K). ERRα knockdown in HL-60 also induced mitochondria-associated apoptosis (Additional file 3: Fig. S2L and M). Our data suggest that blockade of ERRα can induce apoptotic cell death in AML cells.

Lastly, we tested the effects of ERRα inhibition using in vivo xenograft mouse models. First, we evaluated the effect of tumor progression depending on ERRα expression using two different AML xenograft mouse models (Fig. 2J and K; heterotopic and orthotopic murine models of AML, respectively). In NOD/SCID mice, the tumor growth of subcutaneously injected KG1α transduced with shERRα (shERRα-KG1α) was significantly impeded, when compared with that of nonspecific shRNA-transduced KG1α cells (shNS-KG1α) (Fig. 2J). In addition, the survival rates were significantly increased in the NOD/SCID/IL2Rγ^null^ (NIG) mice intravenously engrafted by shERRα-KG1α cells, compared with those engrafted with shNS-KG1α (Fig. 2K). The leukemic burden of the bone marrow was significantly decreased in the XCT-790-treated HL-60-transplanted NOD/SCID mice than those in the vehicle-treated group (Fig. 2L); however, there were no differences of body weights between the vehicle- and XCT-790-treated groups (Additional file 3: Fig. S2N). Together, targeting ERRα promotes antileukemic effects through suppression of mtOXPHOS and inducing apoptotic cell death of AML cells. Considering the long-lasting interest of ERRα action on the solid cancers [12], the current data provide new insights into the role of ERRα as a therapeutic target in hematologic cancers.

## Supplementary Information

**Additional file 1.** **Supplementary Data 1.** ERRα target genes in CCLE AML cell lines.

**Additional file 2.** **Supplementary Data 2.** Differentially expressed genes between control and XCT-790 treated cells.

**Additional file 3.** Supplementary figures and tables.

## Data Availability

Detailed description of the data and methods used in this study is available in Supplementary Information. Other information related with this study would be available upon request to corresponding authors.

## Abbreviations

AML: Acute myeloid leukemia
CCLE: Cancer cell line encyclopedia
DEG: Differentially expressed genes
FDR: False discovery rate
LRT: Log-rank test
OCR: Oxygen consumption rate
OR: Odd ratio
OXPHOS: Oxidative phosphorylation
qRT-PCR: Real-time quantitative PCR
TCGA: The cancer genome atlas
