# Supplementary material for: Targeting ERRα promotes cytotoxic effects against acute myeloid leukemia through suppressing mitochondrial oxidative phosphorylation
Source: J Hematol Oncol. 2022 Oct 26;15:156. doi: 10.1186/s13045-022-01372-7 (PMC9597966; doi:10.1186/s13045-022-01372-7)
Supplement: Supplementary file 3 — Additional file 3. Supplementary figures and tables. [file 13045_2022_1372_MOESM3_ESM.pdf]

Supplementary Information for

Targeting ERRα promotes cytotoxic effects against acute myeloid leukemia through suppressing mitochondrial oxidative phosphorylation

Running title: ERRα inhibition for antileukemic responses in AML

Wonhyoung Seo 1,2 • Seungyeul Yoo 6 • Yi Zhong 6 • Sang-Hee Lee 8 • Soo-Yeon Woo 9 • Hee-Seon Choi 9 • Minho Won 10 • Taylor Roh 1,2 • Sang Min Jeon 1,2 • Kyeong Tae Kim 1,2 • Prashanta Silwal 1,2 • Min Joung Lee 3 • Jun Young Heo 3 • Nathan Lawlor 6 • Sup Kim 11 • Dongjun Lee 9 • Jin-Man Kim 1,2,4 • Ik-Chan Song 5 • Jun Zhu 6,7 • Eun-Kyeong Jo 1,2

These authors contributed equally: Wonhyoung Seo, Seungyeul Yoo

These authors jointly supervised this work: Eun-Kyeong Jo, Jun Zhu, Ik-Chan Song

Affiliations:

1. Department of Medical Science, Chungnam National University College of Medicine, Daejeon, South Korea
2. Infection Control Convergence Research Center; Chungnam National University College of Medicine, Daejeon, South Korea
3. Department of Biochemistry, Chungnam National University College of Medicine, Daejeon, South Korea
4. Department of Pathology, Chungnam National University College of Medicine, Daejeon, South Korea
5. Division of Hematology/Oncology, Department of Internal Medicine, Chungnam National University College of Medicine, Daejeon, South Korea
6. Sema4, Stamford, CT, USA
7. Tisch Cancer Institute, Icahn School of Medicine at Mount Sinai, New York, NY, USA
8. Center for Research Equipment, Korea Basic Science Institute, Cheongju, South Korea
9. Department of Convergence Medicine, School of Medicine, Pusan National University, Yangsan, South Korea
10. Biotechnology Process Engineering Center, Korea Research Institute of Bioscience & Biotechnology, Cheongju, South Korea
11. **11.** Department of Radiation Oncology, Chungnam National University Hospital, Daejeon, South Korea

Supplementary Materials and Methods

Patient samples and cell preparation

The study is approved by IRB (CNUH2018-08-013-012) of Chungnam National University [CNU] Hospital. Bone marrow or peripheral blood samples from 25 AML patients (age = 67 $\pm$ 15.1, sex: female=9 (36%) (Supplementary Table 3) that were collected for diagnostic purposes were obtained for experiments after written informed consent. All experiments were conducted as per the declaration of Helsinki. Human blast cells and mononuclear cells were isolated by Ficoll-Paque gradient ultracentrifugation (Stemcell)[1-3]; isolated mononuclear cells were further incubated with RBC lysis buffer (Invitrogen) to remove the red blood cells. The acquired cells were frozen in Cell freezing medium (Nacalai tesque, Kyoto, Japan) and kept in liquid nitrogen. Before purification of the blast cells, the percentage of blasts in the patient's samples was determined by flow cytometry and morphologic features. All patient-derived blast cells were cultured in 10% FBS in presence of 5% supernatant of the 5637 bladder cancer cell line (to supply interleukin-1 beta (IL-1β), granulocyte-macrophage colony-stimulating factor (GM-CSF), granulocyte colony-stimulating factor (G-CSF), macrophage colony-stimulating factor (M-CSF), and stem cell factor (SCF))[4-6].

Cell culture

THP-1, HL-60, and 5637 cell lines were purchased from the Korean Cell Line Bank (Seoul, Korea), and KG1α was obtained from the Heo Lab at CNU (Daejeon, Korea). All cells were cultured in RPMI 1650 medium (Lonza) supplemented with 10% (20% for HL-60) fetal bovine serum (Gibco) and 50U/ml penicillin/streptomycin at 37°C in a humidified 5% CO_2 incubator.

Immunohistochemistry

The bone marrow of patients with AML or non-hematologic malignancy were acquired from CNU Hospital. The preparation and review of bone marrow slides were conducted based on the IRB CNUH2018-08-013-012. The expression of ERRα in the patient's bone marrow was assessed by immunohistochemistry on paraffin-embedded tissue sections (slide thickness = 3 μm) using the monoclonal ERRα antibody (Cell Signaling Technology, 13826S).

Publicly available AML gene expression datasets

Multiple publicly available gene expression datasets were used in this study; CCLE AML cell lines RNA-Seq; the LM22[7] matrix of reference gene expression of immune cells; multiple independent microarray datasets from healthy controls and AML patients ((GSE42519)[8], GSE63270[9], E-MTAB-220[10], GSE9476[11], and (GSE71014)[12]); and TCGA AML RNA-Seq with survival information[13]. Gene expression data of AML cell lines (35 RNA-Seq and 32 microarray) were downloaded from CCLE database (https://depmap.org/portal/download/). The LM22[7] matrix of reference gene expression of immune cells was downloaded (https://cibersort.stanford.edu/download.php). A total of 11275 genes were used for the quantile normalization of immune cells and AML cell lines. Three independent microarray datasets were used to compare *ERRα* expression in hematopoietic cells from 38 healthy donors and leukemia blasts from 26 AML patients; GSE63270[9]: bone marrows from 7 healthy donors and 21 AML patients; E-MTAB-220[10]: bone marrows from 10 healthy donors and 33 AML patients; GSE9476[11]: bone marrows from 28 healthy donor and leukemic blasts from 26 AML patients. Microarray data for the myeloid arm of the hematopoietic systems were used to analyze *ERRα* expression in hematopoietic stem and progenitor cells. RNA-Seq data of 173 AML patients with survival information[13] were downloaded from the TCGA data portal (https://portal.gdc.cancer.gov/projects/TCGA-LAML). In addition, a set of microarray profiles of 104 AML patients (GSE71014)[12] was used as an independent cohort for survival analysis.

ERRα target genes

The predicted ERRα binding sites were retrieved from a deposited ChIP result of K562, a chronic myeloid cell line (http://encodec.encodeproject.org/#data). Total 7831 ERRα binding targets identified in a proximal TSS-based network[14] were further intersected with $ERR\alpha$ co-expressed genes in AML cell lines (p<0.05), and then separated into 613 $ERR\alpha^{+}$ and 256 $ERR\alpha^{-}$ genes based on co-expression direction.

ERRα activity score estimated with filtered ERRα target genes

While 61% of ERRα target genes showed similar co-expression patterns with ERRα between cell lines and TCGA bulk tumors, some showed weak, or even inverse correlations with $ERR\alpha$ in bulk tumors indicating tumor microenvironment effect within bulk tumors[15]. Hence, the ERRα target genes defined in AML cell lines were first filtered based on the co-expression patterns in TCGA AML samples. With a cutoff at Pearson correlation $p<10^{-6}$, ERRα co-expressed genes in TCGA AML samples significantly overlapped with the ERRα binding target genes in K562 ChIP for both directions (Fisher's Exact Test [FET] Odd-Ratio [OR] > 2). The ERRα target gene sets from CCLE samples were further intersected with $ERR\alpha$ co-expressed genes in TCGA samples, and we selected 145 $ERR\alpha^{+}$ and 44 $ERR\alpha^{-}$ genes commonly identified in both cell lines and bulk tumors. (FET ORs = 5.5 and 4.2, $p = 1.5 \times 10^{-48}$ and 3.2×10^{-13} for $ERR\alpha^{+}$ and $ERR\alpha^{-}$ genes, respectively; Fig. S1e-f). For each sample, the resulting z-scores of the 145 $ERR\alpha^{+}$ and 44 $ERR\alpha^{-}$ genes from bulk tissue gene expression datasets (TCGA[13] and GSE71014[12]) were averaged. The final ERRα activity score was defined as $ERR\alpha\ activity_{score} = ERR\alpha^{+}_{score} - ERR\alpha^{-}_{score}$.

Survival analysis

Two independent AML cohorts (TCGA and GSE71014) were used for survival analysis. Hazard ratios with 95% confidence intervals of $ERR\alpha$ expression and ERRα activity scores were estimated using a multivariate cox regression model with age and sex as covariates $Survival \sim age + sex + ERRa\ (expression\ or\ activity\ score)$. In addition, samples in each cohort were classified into three groups based on the mean and standard deviation of ERRα expression or activity scores (low: $ERR\alpha$ < mean – sd, medium: mean – sd < $ERR\alpha$ < mean + sd, high: mean + sd < $ERR\alpha$), then we compared survival rates across the groups using log-rank test (LRT). The coxph() function from an R package "survival" was used to perform the survival analysis.

RNA-Seq analysis

Total RNA was isolated using Trizol reagent (Invitrogen). Quality of extracted RNA was assessed by Agilent 2100 bioanalyzer (Agilent Technologies, Amstelveen, Netherlands), and RNA quantification was measured using an ND-2000 Spectrophotometer (Thermo Inc., DE, USA). Libraries were constructed from total RNA using the NEBNext Ultra II Directional RNA-Seq Kit (NEW ENGLAND BioLabs, Inc., UK). The mRNA was isolated using the Poly(A) RNA Selection Kit (LEXOGEN, Inc., Austria) and transformed into the cDNA and shearing. Indexing was performed using the Illumina indexes. High-throughput sequencing was performed as paired-end 100 sequencing using NovaSeq 6000 (Illumina, Inc., USA). Read counts per RefGene symbol of the USCS database were estimated using the htseq-count function in SAMtools[16]. DEseq2[17] was used to identify differentially expressed genes between controls and XCT-790 treated cells (FDR<0.01 & FC>1.5).

Single-cell RNA sequencing analysis

For the single-cell RNA-Seq data of three AML patients from van Galen et al. (GSE116256)[18], the processed data with malignancy information (normal vs cancer) of individual cells were downloaded.

For a validation data, cells from an AML-patient in CNU hospital (a female, >80 years old, leukocyte = 83,659/uL, Leukemia %= 90) were collected. Library construction was performed using the 10X Chromium Single-cell 3' Reagent Kits v3.1, and the library was sequenced by Illumina NovaSeq 6000 platform. Preliminary sequencing data were converted to FASTQ files using Cell Ranger. We followed the 10x Genomics standard seq methodology to trim the barcode and unique molecular identifier (UMI) ends to 26 bp and the mRNA end to 98 bp. The FASTQ data were then aligned to the human reference genome (GRCh38). Subsequently, we applied Cell Ranger for preliminary data analysis and generated a file that contained a barcode table, a gene table, and a gene expression matrix. Using the R package Seurat (v4.0.4)[19], we applied two quality measures to the raw gene-cell-barcode matrix for individual cells: mitochondrial genes (<10% and feature count>500). Genes not expressed in any cells were removed from further normalization (scale.factor=10000). The top 4000 most varying genes were selected, then UMI counts for the genes in each cell were transformed in the log2 scale. Then, the principal components were computed and the top 10 PCs were selected using the ElbowPlot() of Seurat. Cell clustering were performed using the FindClusters() and RunUMAP() functions, respectively. The copy number alterations of individual cells were determined by CopyKat[20]. CopyKat took the raw count gene expression matrix as input and predicted the status of each cell into aneuploid or diploid.
The processed data of normal PBMC single-cell from two publicly available cohorts, Seurat PBMC 3k PMBC (https://satijalab.org/seurat/articles/pbmc3k_tutorial.html) and Human Cell Atlas CZI.PMBC data (https://data.humancellatlas.org/explore/projects/efea6426-510a-4b60-9a19-277e52bfa815/project-matrices), were downloaded.

Functional enrichment analysis for apoptosis quantification and cell cycle analysis

For functional annotations, Hallmark gene sets, KEGG pathways, and GO terms in Molecular Signatures Database (MSigDB) were used[21]. One side FET was performed to estimate the odd-ratio and p-value of the overlap between two gene sets[22]. A single sample gene set enrichment analysis (ssGSEA) was performed to estimate normalized enrichment score (NES) of OXPHOS genes[23].

RNA extraction and real-time quantitative PCR (qRT-PCR)

RNA extraction and real-time quantitative PCR were performed using TRizol reagent (Invitrogen), Reverse transcriptase kit (Elpis, Daejeon, South Korea), and SYBR Green PCR Kits (Qiagen) in the Real-time PCR cycler Rotor-Gene Q 2plex system (Qiagen)[24, 25]. Relative mRNA expression of various target genes was calculated through the $2^{-\Delta\Delta Ct}$ method and the $2^{-\Delta\Delta Ct}$ value of human GAPDH was used as a control gene, and each RNA expression is displayed as relative fold changes.

Western blot

Cells were harvested and lysed in 4°C for 2 hours using Radioimmunoprecipitation assay buffer (RIPA buffer, LPS solution, Korea) with protease inhibitor cocktails (Roche). The equal amounts of proteins were mixed with SDS sample buffer (Elipis biotech, Korea) and were boiled for 10 min. Then the samples were separated by SDS-polyacrylamide gel electrophoresis and transferred to nitrocellulose membrane or polyvinylidene fluoride membrane (Millipore). Membranes were blocked in 5% skimmed milk in 0.1% TBST for 30 min and probed with the specific primary and peroxidase-conjugated secondary antibodies. Signals were visualized by using an ECL solution (Millipore) in a chemiluminescence device (UVitec, UK).

Flow cytometry analysis

The quantitation of apoptosis was confirmed by flow cytometry (FACSCanto II or NovoCyte flow cytometer) using FITC Annexin V apoptosis detect Kit (BD Bioscience) or annexin V-APC (BD Bioscience) and propidium iodide (PI)(BD Bioscience, USA). To assess the cell cycle (shNS vs. shERRα), immunofluorescent staining of incorporated bromodeoxyuridine (BrdU) and flow cytometric analysis were performed using the FITC BrdU Flow Kit (BD Bioscience). PI was used

for analyzing the fraction of cell cycle in wild-type and ERRα-knockout cells through the PE channel.

CRISPR-Cas-9 mediated ERRα knockout of KG1α cells

The guide oligonucleotide targeting ERRα (GCAACTAGTGCGGCGGCTGC) was synthesized and inserted into the px330- puro vector (Dr. Macfarlan, NICHD, NIH, USA) through the standard protocol[25] and the gDNA sequences were designed. The constructed vectors were transfected into cells using electroporation according to the manufacturer's instructions (Nucleofector, Lonza). After 24h of transfection of PX330, the cells were exposed to 2 μg/mL puromycin (Gibco) for one day, and a single-cell clone was selected.

Stable AML cell lines with ERRα knockdown

All transfections were performed in HEK293T cells using lipofectamine 3000 (Thermo Fisher Scientific) with 3rd generation lentiviral packaging system. Viral supernatant was collected 24 h and 52 h post-transfection. For transduction of AML cells, spinoculation was performed at room temperature by centrifuging the cells at 200x g for 60 min in presence of polybrene (10 μg/mL). After 72 h transduction, puromycin selection (1.0~2.0 μg/mL) was done for 5~7 days. The shRNA plasmids used in this study; Lentivirus with nonspecific scramble shRNA (SHC016) and ERRα targeted shRNA (NM_004451.3-1881s21c1, NM_004451.3-757s21c1).

Cell viability, cell proliferation assay & IC_{50} measurements

Cells were seeded into 96-well plates at 70~80% confluency and viability assays were performed by adding 10% CCK-8 (DOJINDO, Japan) for 2~4 hours. A microplate spectrophotometer was used to measure the optical density [O.D] at 450nm. To quantify the cell proliferation, cells were seeded in 96 well plates and then incubated for up to five days. The absolute amounts of live cells were determined by counting the cells in a hemocytometer using the trypan blue exclusion method and converted to percentage viability versus the vehicle control. IC_{50} values was obtained from non-linear regression of '[Inhibitor] vs. normalized response - variable slope' in GraphPad Prism v8.0.

Mitochondrial Respiration

Oxygen consumption rate (OCR, pmol/min/unit) was determined using the XF24 (THP-1: 5 x 10^5 cells/mL) and XFe96 (KG1α: 1 x 10^5 cells/mL) analyzer (Agilent). Oxygen consumption was monitored over time, beginning with baseline conditions, and progressing through serial injection of Oligomycin (an ATPase inhibitor, final concentration 2 μg/ml), CCCP (an uncoupler, final concentration 5 μM), and Rotenone (mitochondrial complex I inhibitor, final concentration 2 μM). Information about materials, primers, and reagents used in this study are in Supplementary Table 4.

Electron microscopy and morphology

Cells were washed in PBS before fixing with 3% glutaraldehyde for 3 h in 0.1 M sodium cacodylate buffer (pH 7.2) containing 0.1% $CaCl_2$. Then the samples were post-fixed for 2 h with 1% osmium tetroxide in 0.1 M sodium cacodylate buffer with 0.1% $CaCl_2$, and then were washed with cold distilled water and slowly dehydrated at 4°C using a series of ethanol concentrations and propylene oxide. Embed-812 (EMS; 14120) was used to embed the samples, which were then cured at 60°C for 36 h. The ultrathin sections (70–80 nm) were cut with a diamond knife mounted on formvar-coated slot grids using a ULTRACUT UC7 ultramicrotome (Leica, Germany). Sections were stained with 4% uranyl acetate for 10 min and with lead citrate for 7 min. A Bio-High Voltage EM system (JEM-1400 Plus and JEM-1000 BEF; JEOL Ltd., Tokyo, Japan) was used to scan stained sections. For morphometric analysis of mitochondrial cristae, cristae widths were measured

through ImageJ on TEM images containing measurable cristae width and 10 mitochondrial sections were counted to measure the length of at least 18 cristae[26].

Animal experiments and tumor xenograft

The *in vivo* experiments were reviewed and approved by the Institutional Animal Care and Use Committee (CNUH-020-A0054, CNUH-2022-A0010-00, and PNU-2022-0141). All animals (6~8 weeks, male) were housed in a specific pathogen-free environment. Except the experiment of mouse survival rates, NOD/SCID mice, purchased from KOATECH (Pyeongtaek, Gyeonggi, Korea), were used. To evaluate the rate of tumor progression depending on ERRα status, KG1α cells transduced with shRNA targeting ERRα or control were subcutaneously injected in NOD/SCID mice for measuring the gross tumor mass (5 x 10^6 cells in 100 μL of Hank's Balanced Salt Solution). After tumor mass was detected, the volumes of tumors were measured every 3 days and estimated by the tumor length (*a*) and width (*b*) measured by a digital caliper using the formula $V = 1/2(a \times b^2)$. To evaluate the therapeutic potency of XCT-790, HL-60 cells were intravenously injected (4 x 10^6 cells in 100 μL of Hank's Balanced Salt Solution) through the tail vein in NOD/SCID mice 24 h after radiation (2.5 Gy). After 7 days of xenotransplantation, XCT-790 (8 mg/kg) was treated via the intraperitoneal route every 2 days for 3 weeks. The body weights of mice were measured every 2 days until sacrificed. The engrafted HL-60 cells in bone marrow of mice were acquired and analyzed through flow cytometry with anti-human CD45 and anti-murine CD45 antibodies.

For the measurement of mouse survival rates after xenotransplantation with KG1α cells, NOD/SCID/IL2Rγ^{null} (NIG) mice purchased from GHbio (Daejeon, Korea), which have the same phenotype with NSG (NOD/SCID/IL2Rγ^{null})[27], were used. KG1α cells were injected through tail vein in NIG mice for survival analysis (4 x10^6 cells in 100 μL of Hank's Balanced Salt Solution). The number of surviving mice was observed until 63 days after xenotransplantation.

Statistical analysis for *in vitro* and *in vivo* data

The statistical analysis for *in vitro* and *in vivo* data was done with the SPSS or Prism software (GraphPad). According to the normality test, a two-tailed t-test or non-parametric test was used to compare the two conditions. $P < 0.05$ (*), $P < 0.01$ (**) and $P < 0.001$ (***) were used to determine statistically significant differences.

REFERENCES:

1. Andresen V, Erikstein BS, Mukherjee H, et al. Anti-proliferative activity of the NPM1 interacting natural product avrainvillamide in acute myeloid leukemia. Cell Death Dis 2016;7:e2497.
2. Wei TW, Wu PY, Wu TJ, et al. Aurora A and NF-kappaB Survival Pathway Drive Chemoresistance in Acute Myeloid Leukemia via the TRAF-Interacting Protein TIFA. Cancer Res 2017;77:494-508.
3. Bruserud O, Gjertsen BT, Foss B, et al. New strategies in the treatment of acute myelogenous leukemia (AML): in vitro culture of aml cells--the present use in experimental studies and the possible importance for future therapeutic approaches. Stem Cells 2001;19:1-11.
4. Quentmeier H, Zaborski M, Drexler HG. The human bladder carcinoma cell line 5637 constitutively secretes functional cytokines. Leuk Res 1997;21:343-50.
5. Niu X, Wang G, Wang Y, et al. Acute myeloid leukemia cells harboring MLL fusion genes or with the acute promyelocytic leukemia phenotype are sensitive to the Bcl-2-selective inhibitor ABT-199. Leukemia 2014;28:1557-60.
6. Qiao X, Ma J, Knight T, et al. The combination of CUDC-907 and gilteritinib shows promising in vitro and in vivo antileukemic activity against FLT3-ITD AML. Blood Cancer J 2021;11:111.
7. Newman AM, Liu CL, Green MR, et al. Robust enumeration of cell subsets from tissue expression profiles. Nat Methods 2015;12:453-7.
8. Rapin N, Bagger FO, Jendholm J, et al. Comparing cancer vs normal gene expression profiles identifies new disease entities and common transcriptional programs in AML patients. Blood 2014;123:894-904.
9. Jung N, Dai B, Gentles AJ, et al. An LSC epigenetic signature is largely mutation independent and implicates the HOXA cluster in AML pathogenesis. Nat Commun 2015;6:8489.
10. Beghini A, Corlazzoli F, Del Giacco L, et al. Regeneration-associated WNT signaling is activated in long-term reconstituting AC133bright acute myeloid leukemia cells. Neoplasia 2012;14:1236-48.
11. Stirewalt DL, Meshinchi S, Kopecky KJ, et al. Identification of genes with abnormal expression changes in acute myeloid leukemia. Genes Chromosomes Cancer 2008;47:8-20.
12. Chuang MK, Chiu YC, Chou WC, et al. An mRNA expression signature for prognostication in de novo acute myeloid leukemia patients with normal karyotype. Oncotarget 2015;6:39098-110.
13. Cancer Genome Atlas Research N, Ley TJ, Miller C, et al. Genomic and epigenomic landscapes of adult de novo acute myeloid leukemia. N Engl J Med 2013;368:2059-74.
14. Zhang J, Lee D, Dhiman V, et al. An integrative ENCODE resource for cancer genomics. Nat Commun 2020;11:3696.

15. Lamble AJ, Kosaka Y, Laderas T, et al. Reversible suppression of T cell function in the bone marrow microenvironment of acute myeloid leukemia. Proc Natl Acad Sci U S A 2020;117:14331-14341.
16. Li H, Handsaker B, Wysoker A, et al. The Sequence Alignment/Map format and SAMtools. Bioinformatics 2009;25:2078-9.
17. Love MI, Huber W, Anders S. Moderated estimation of fold change and dispersion for RNA-seq data with DESeq2. Genome Biol 2014;15:550.
18. van Galen P, Hovestadt V, Wadsworth Ii MH, et al. Single-Cell RNA-Seq Reveals AML Hierarchies Relevant to Disease Progression and Immunity. Cell 2019;176:1265-1281 e24.
19. Hao Y, Hao S, Andersen-Nissen E, et al. Integrated analysis of multimodal single-cell data. Cell 2021;184:3573-3587 e29.
20. Gao R, Bai S, Henderson YC, et al. Delineating copy number and clonal substructure in human tumors from single-cell transcriptomes. Nat Biotechnol 2021;39:599-608.
21. Liberzon A, Subramanian A, Pinchback R, et al. Molecular signatures database (MSigDB) 3.0. Bioinformatics 2011;27:1739-40.
22. Yoo S, Sinha A, Yang D, et al. Integrative network analysis of early-stage lung adenocarcinoma identifies aurora kinase inhibition as interceptor of invasion and progression. Nat Commun 2022;13:1592.
23. Alvarez MJ, Shen Y, Giorgi FM, et al. Functional characterization of somatic mutations in cancer using network-based inference of protein activity. Nat Genet 2016;48:838-47.
24. Kim SY, Yang CS, Lee HM, et al. ESRRA (estrogen-related receptor alpha) is a key coordinator of transcriptional and post-translational activation of autophagy to promote innate host defense. Autophagy 2018;14:152-168.
25. Kim S, Lee JY, Shin SG, et al. ESRRA (estrogen related receptor alpha) is a critical regulator of intestinal homeostasis through activation of autophagic flux via gut microbiota. Autophagy 2021;17:2856-2875.
26. Kaurov I, Vancova M, Schimanski B, et al. The Diverged Trypanosome MICOS Complex as a Hub for Mitochondrial Cristae Shaping and Protein Import. Curr Biol 2018;28:3393-3407 e5.
27. Bak I, Kim DJ, Kim HC, et al. Two base pair deletion in IL2 receptor gamma gene in NOD/SCID mice induces a highly severe immunodeficiency. Lab Anim Res 2020;36:27.

Supplementary Figures

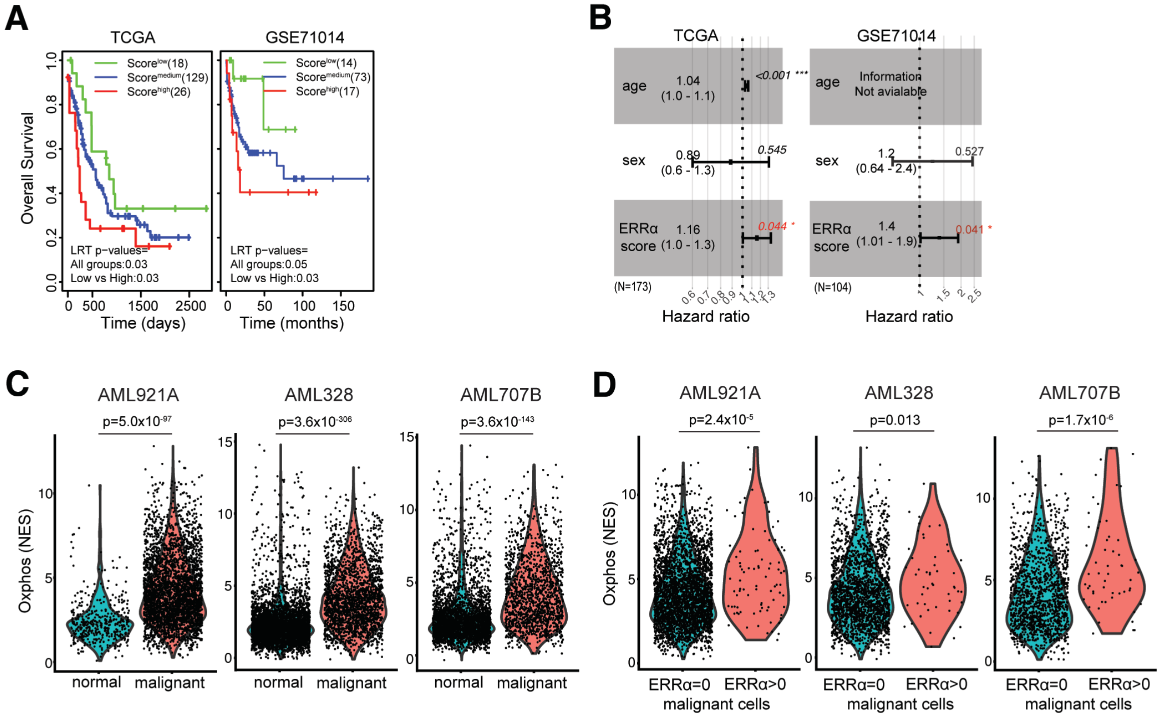

Fig. S1. *ERRα* regulates OXPHOS in AML cells

A. KM plot showing survival probability differences among patients stratified by mean and standard deviation of *ERRα* activity scores into low, medium, and high groups. *P* values were calculated by log-rank test (LRT). **B.** Hazard ratios and 95% confidence intervals of ERRα activity score measured by a multivariate cox regression model using age and sex as covariates. Center dots indicate Hazard ratios and error bars indicate upper and lower 95% confidence intervals. Significant *p* values were marked in red. **C.** Comparison of HALLMARK OXPHOS pathway activity measured by normalized enrichment scores (NES) between individual malignant and normal cells in bone marrows in van Galen et al.[18]. **D.** Comparison of OXPHOS pathway activity between individual *ERRα* expressing malignant cells and other malignant cells.

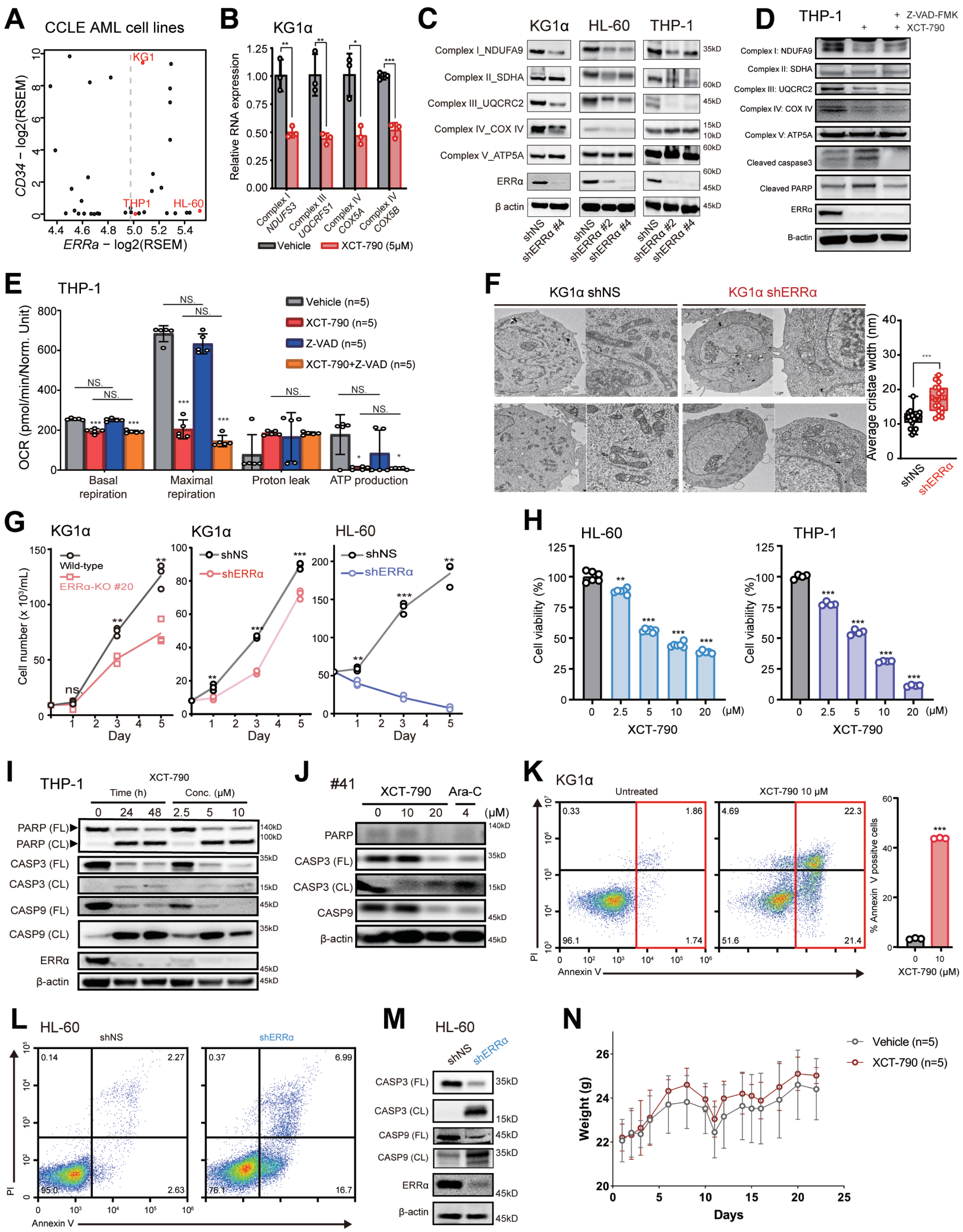

Fig. S2. ERRα inhibition induces apoptotic cell death through mtOXPHOS suppression in AML cells

A. Expression of *ERRα* and *CD34* in CCLE AML cell line. A red dashed line indicates a median value of ERRα among 35 CCLE AML cell lines. **B.** Relative expression of *NDUFS3, UQCRFS1, COX5A,* and *COX5B* in KG1α cells significantly downregulated by XCT-790 treatment (5 μM for 24 h, mean ± SD, n = 3). **C.** Western analysis of mtOXPHOS complexes in KG1α, HL-60 and THP-1 cells by transduction with either lentiviral shRNA targeting ERRα (shERRα) or non-targeting control shRNA lentivirus (shNS). **D.** Western analysis of mtOXPHOS complexes by XCT-790 treatment (5 μM) in THP-1 cells in the presence or absence of Z-VAD-FMK (20 μM). Total incubation time was 48 h. **E.** Bar-plot indicating mitochondrial respiration-related markers (baseline respiratory capacity, maximum respiratory capacity, proton leak, and ATP turnover) based on the data of oxygen consumption rates in THP-1 cells (one-way ANOVA, mean ± SD, n = 5). **F.** Representative electron microscope of ERRα-knockdowned KG1α cells by transduction with either shERRα (KG1α shERRα) or shNS (KG1α shNS). Scale bars, 1 μM and 0.2 μM. Quantification of the cristae width from 20 randomly selected mitochondria (right). *P* values were calculated by two-tailed *t* test. **G.** Quantification of cell viability of AML cells by either ERRα knockout (KO) by CRISPR-Cas9-mediated gene deletion (KG1α ERRα-KO #20 vs wild type, left) or by knocking-down with shERRα (KG1α and HL-60, middle and right, respectively). Cell viability was determined by either CCK8 assay (for KG1α) and trypan blue exclusion assay (for HL-60). *P* values were calculated by two-tailed *t* test (two-tailed *t* test, mean ± SD, n = 3). **H.** CCK8 assay in HL-60 and THP-1 cells by XCT-790 treatment at the indicated doses for 48 h. *P* values were calculated by one-way ANOVA. **I** and **J**. Western blots of apoptotic proteins in (**I**) THP-1 cells by XCT-790 treatment at 5 μM (lanes 2 and 3, for 24 and 48 h, respectively) and at the indicated doses (lanes 4-6, for 48 h); and (**J**) in AML patient-derived cells (#41) with XCT-790 or Ara-C treatment for 72 h. **K** and **L**. Flow cytometric analysis of apoptotic cell death using Annexin V-FITC/PI staining in KG1α cells with XCT-790 treatment (for **K**; 10 μM for 72 h) and HL-60 cells by transduction with either shERRα or shNS at 7 days (for **L**). Quantitative analysis of

Annexin V-positive cell fractions (%; right). *P* value was calculated by two-tailed *t* test (mean ± SD, n = 3). **M.** Western analysis of apoptotic proteins in HL-60 cells at the same conditions of **L**. **N.** A comparison of body weight between the vehicle-treated and XCT-790-treated groups of HL-60-transplanted NOD/SCID mice. HL-60 cells were engrafted through tail vein ($4x10^6$ cells/mice) 24 h after radiation (2.5 Gy). Starting from 1 week after transplantation (Day 0 on the X-axis), XCT-790 (8 mg/kg) was treated via the intraperitoneal route once every 2 days for 3 weeks. Data indicate mean ± SD. $P < 0.05$ (*), $P < 0.01$ (**), and $P < 0.001$ (***) were used to determine statistically significant differences.

Supplementary Tables

Supplementary Table 1. Gene sets enriched within the ERRα target genes in AML cell lines

ERRα+ target genes

HALLMARK

Geneset	Size	Overlap	OddRatio	p-value	q-value
HALLMARK_OXIDATIVE_PHOSPHORYLATION	184	35	9.48	9.74E-21	4.87E-19
HALLMARK_MYC_TARGETS_V2	58	14	12.45	1.47E-10	3.69E-09
HALLMARK_DNA_REPAIR	148	15	4.41	5.90E-06	9.83E-05
HALLMARK_ADIPOGENESIS	186	16	3.68	2.35E-05	0.000293288
HALLMARK_MYC_TARGETS_V1	194	16	3.51	3.93E-05	0.000392818
HALLMARK_E2F_TARGETS	195	13	2.78	0.001521019	0.012675161
HALLMARK_G2M_CHECKPOINT	190	12	2.62	0.003538363	0.025274021

KEGG pathways

Geneset	Size	Overlap	OddRatio	p-value	q-value
KEGG_ALZHEIMERS_DISEASE	145	19	5.93	5.39E-09	1.00E-06
KEGG_PARKINSONS_DISEASE	107	16	6.89	1.28E-08	1.19E-06
KEGG_HUNTINGTONS_DISEASE	157	19	5.41	2.03E-08	1.26E-06
KEGG_OXIDATIVE_PHOSPHORYLATION	108	14	5.82	6.32E-07	2.94E-05
KEGG_APOPTOSIS	82	9	4.78	0.000235393	0.008756617
KEGG_SPLICEOSOME	123	11	3.81	0.000309102	0.009582173

GO BP

Geneset	Size	Overlap	OddRatio	p-value	q-value
GO_MITOCHONDRIAL_GENE_EXPRESSION	151	36	12.68	7.34E-25	5.53E-21
GO_MITOCHONDRIAL_TRANSLATION	128	33	14.01	4.57E-24	1.72E-20
GO_NCRNA_METABOLIC_PROCESS	409	54	6.29	2.33E-23	5.85E-20
GO_NCRNA_PROCESSING	342	49	6.88	7.38E-23	1.39E-19
GO_RIBOSOME_BIOGENESIS	269	42	7.54	3.38E-21	5.10E-18
GO_RIBONUCLEOPROTEIN_COMPLEX_BIOGENESIS	393	49	5.85	3.30E-20	4.15E-17
GO_RNA_PROCESSING	845	73	3.98	4.93E-20	5.30E-17
GO_MITOCHONDRION_ORGANIZATION	469	51	5.01	2.20E-18	2.07E-15
GO_MITOCHONDRIAL_TRANSLATIONAL_TERMINATION	87	23	14.26	1.96E-17	1.64E-14
GO_TRANSLATIONAL_TERMINATION	102	24	12.22	7.33E-17	5.52E-14
GO_TRANSLATIONAL_ELONGATION	127	26	10.26	1.47E-16	1.00E-13
GO_PEPTIDE_BIOSYNTHETIC_PROCESS	597	54	4.08	6.96E-16	4.37E-13
GO_AMIDE_BIOSYNTHETIC_PROCESS	726	60	3.72	1.02E-15	5.90E-13
GO_RRNA_METABOLIC_PROCESS	202	29	6.69	4.74E-14	2.55E-11
GO_TRNA_METABOLIC_PROCESS	157	25	7.52	2.46E-13	1.23E-10
GO_PEPTIDE_METABOLIC_PROCESS	723	55	3.37	5.11E-13	2.41E-10
GO_CELLULAR_PROTEIN_CONTAINING_COMPLEX_ASSEMBLY	888	62	3.09	6.83E-13	3.02E-10
GO_MITOCHONDRIAL_RESPIRATORY_CHAIN_COMPLEX_ASSEMBLY	89	19	10.70	8.23E-13	3.44E-10
GO_CELLULAR_AMIDE_METABOLIC_PROCESS	959	64	2.94	2.08E-12	8.25E-10
GO_TRNA_PROCESSING	119	21	8.47	2.62E-12	9.86E-10
GO_CELLULAR_PROTEIN_COMPLEX_DISASSEMBLY	205	26	5.77	1.75E-11	6.28E-09
GO_RIBOSOMAL_LARGE_SUBUNIT_BIOGENESIS	65	15	11.76	6.49E-11	2.22E-08
GO_ATP_SYNTHESIS_COUPLED_ELECTRON_TRANSPORT	89	17	9.28	8.64E-11	2.83E-08
GO_RNA_MODIFICATION	147	21	6.58	1.66E-10	5.21E-08

GO_PROTEIN_CONTAINING_COMPLEX_DISASSEMBLY	303	30	4.38	2.59E-10	7.79E-08
GO_NADH_DEHYDROGENASE_COMPLEX_ASSEMBLY	61	14	11.66	3.05E-10	8.84E-08
GO_OXIDATIVE_PHOSPHORYLATION	115	18	7.29	7.40E-10	2.06E-07
GO_METHYLATION	306	29	4.16	1.43E-09	3.62E-07
GO_TRNA_MODIFICATION	80	15	9.04	1.46E-09	3.62E-07
GO_RESPIRATORY_ELECTRON_TRANSPORT_CHAIN	106	17	7.50	1.49E-09	3.62E-07
GO_MITOCHONDRIAL_TRANSMEMBRANE_TRANSPORT	72	14	9.44	3.13E-09	7.37E-07
GO_DNA_TEMPLATED_TRANSCRIPTION_INITIATION	227	24	4.67	4.41E-09	1.01E-06
GO_MITOCHONDRIAL_ELECTRON_TRANSPORT_NADH_TO_UBIQUINONE	53	12	11.42	6.76E-09	1.50E-06
GO_CELLULAR_COMPONENT_DISASSEMBLY	503	37	3.17	9.17E-09	1.97E-06
GO_DNA_TEMPLATED_TRANSCRIPTION_ELONGATION	105	16	7.05	9.69E-09	2.00E-06
GO_PROTEIN_IMPORT_INTO_MITOCHONDRIAL_MATRIX	19	8	28.20	9.85E-09	2.00E-06
GO_MITOCHONDRIAL_TRANSPORT	222	23	4.56	1.36E-08	2.70E-06
GO_CELLULAR_RESPIRATION	170	20	5.25	1.40E-08	2.71E-06
GO_ELECTRON_TRANSPORT_CHAIN	172	20	5.18	1.72E-08	3.23E-06
GO_ESTABLISHMENT_OF_PROTEIN_LOCALIZATION_TO_ORGANELLE	531	36	2.90	1.14E-07	2.09E-05
GO_DNA_METABOLIC_PROCESS	815	47	2.46	1.79E-07	3.21E-05
GO_ATP_METABOLIC_PROCESS	256	23	3.89	1.90E-07	3.32E-05
GO_PROTEIN_LOCALIZATION_TO_MITOCHONDRION	132	16	5.40	2.59E-07	4.33E-05
GO_ENERGY_DERIVATION_BY_OXIDATION_OF_ORGANIC_COMPOUNDS	244	22	3.90	3.28E-07	5.38E-05
GO_PROTEIN_TARGETING_TO_MITOCHONDRION	93	13	6.34	6.59E-07	0.00010555
GO_GENERATION_OF_PRECURSOR_METABOLITES_AND_ENERGY	460	31	2.86	9.86E-07	0.000154672
GO_OXIDATION_REDUCTION_PROCESS	899	48	2.26	1.21E-06	0.000186259
GO_RNA_METHYLATION	69	11	7.38	1.26E-06	0.000189376
GO_PROTEIN_LOCALIZATION_TO_ORGANELLE	874	47	2.28	1.29E-06	0.000190576
GO_PROTEIN_TRANSMEMBRANE_TRANSPORT	57	10	8.27	1.54E-06	0.000223713
GO_PROTEIN_TRANSMEMBRANE_IMPORT_INTO_INTRACELLULAR_ORGANELLE	34	8	11.93	1.69E-06	0.000240034
GO_MACROMOLECULE_METHYLATION	252	21	3.57	2.17E-06	0.000302874
GO_TRNA_METHYLATION	36	8	11.08	2.69E-06	0.000368614
GO_INNER_MITOCHONDRIAL_MEMBRANE_ORGANIZATION	27	7	13.55	3.79E-06	0.000506473
GO_TRANSCRIPTION_ELONGATION_FROM_RNA_POLYMERASE_II_PROMOTER	77	11	6.49	3.83E-06	0.000506473
GO_PROTEIN_COFACTOR_LINKAGE	11	5	32.17	4.25E-06	0.000552232
GO_TRANSCRIPTION_INITIATION_FROM_RNA_POLYMERASE_II_PROMOTER	182	17	4.03	4.39E-06	0.000560408
GO_MITOTIC_CELL_CYCLE	945	47	2.09	1.03E-05	0.001295387
GO_CELL_CYCLE_PHASE_TRANSITION	569	33	2.44	1.11E-05	0.001371576
GO_PROTEIN_IMPORT	180	16	3.81	1.56E-05	0.001865982
GO_MITOCHONDRIAL_MEMBRANE_ORGANIZATION	107	12	4.92	1.80E-05	0.0021212
GO_CELLULAR_RESPONSE_TO_DNA_DAMAGE_STIMULUS	774	40	2.16	2.02E-05	0.002330219
GO_CELLULAR_MACROMOLECULE_CATABOLIC_PROCESS	1086	51	1.97	2.04E-05	0.002330219
GO_NEGATIVE_REGULATION_OF_RNA_BIOSYNTHETIC_PROCESS	1125	52	1.94	2.58E-05	0.002901937
GO_NCRNA_TRANSCRIPTION	94	11	5.15	2.68E-05	0.002971679
GO_TRANSCRIPTION_INITIATION_FROM_RNA_POLYMERASE_I_PROMOTER	36	7	9.34	2.92E-05	0.003182182
GO_REGULATION_OF_CELL_CYCLE	1102	51	1.94	3.00E-05	0.003182182
GO_INTRACELLULAR_PROTEIN_TRANSMEMBRANE_TRANSPORT	49	8	7.56	3.00E-05	0.003182182
GO_NEGATIVE_REGULATION_OF_NUCLEOBASE_CONTAINING_COMPOUND_METABOLIC_PROCESS	1286	57	1.86	3.43E-05	0.003584929
GO_PROTEIN_MODIFICATION_BY_SMALL_PROTEIN_CONJUGATION_OR_REMOVAL	992	47	1.98	3.49E-05	0.003603621
GO_REGULATION_OF_MITOCHONDRIAL_GENE_EXPRESSION	26	6	11.60	3.93E-05	0.004000964

GO_POSITIVE_REGULATION_OF_GENE_EXPRESSION_EPIGENETIC	51	8	7.21	4.05E-05	0.004067873
GO_MITOCHONDRIAL_RNA_METABOLIC_PROCESS	38	7	8.74	4.22E-05	0.004112631
GO_SNRNA_TRANSCRIPTION	66	9	6.13	4.26E-05	0.004112631
GO_PROTEIN_FOLDING	198	16	3.43	5.03E-05	0.004790828
GO_MRNA_METABOLIC_PROCESS	779	39	2.09	5.11E-05	0.004807891
GO_DNA_REPAIR	486	28	2.41	5.71E-05	0.005310654
GO_RIBOSOME_ASSEMBLY	55	8	6.60	7.09E-05	0.006439401
GO_REGULATION_OF_MITOTIC_CELL_CYCLE	598	32	2.23	7.10E-05	0.006439401
GO_RIBONUCLEOPROTEIN_COMPLEX_SUBUNIT_ORGANIZATION	183	15	3.48	7.29E-05	0.006534098
GO_MACROMOLECULE_CATABOLIC_PROCESS	1294	56	1.81	7.64E-05	0.006771781
GO_REGULATION_OF_CELL_CYCLE_PHASE_TRANSITION	420	25	2.49	8.44E-05	0.007389644
GO_REGULATION_OF_GENE_EXPRESSION_EPIGENETIC	275	19	2.90	8.77E-05	0.007594898
GO_TRANSCRIPTION_ELONGATION_FROM_RNA_POLYMERASE_I_PROMOTER	30	6	9.66	9.30E-05	0.00786908
GO_TERMINATION_OF_RNA_POLYMERASE_I_TRANSCRIPTION	30	6	9.66	9.30E-05	0.00786908
GO_NEGATIVE_REGULATION_OF_BIOSYNTHETIC_PROCESS	1401	59	1.76	0.000103107	0.008626587
GO_MITOCHONDRIAL_RESPIRATORY_CHAIN_COMPLEX_IV_ASSEMBLY	20	5	12.87	0.000118037	0.009767263
GO_MATURATION_OF_SSU_RRNA_FROM_TRICISTRONIC_RRNA_TRANSCRIPT_SSU_RRNA_5_8S_RRNA_LSU_RRNA	32	6	8.92	0.000135948	0.011127056
GO_CELLULAR_NITROGEN_COMPOUND_CATABOLIC_PROCESS	541	29	2.23	0.000148833	0.0120507
GO_PROTEIN_DNA_COMPLEX_SUBUNIT_ORGANIZATION	196	15	3.23	0.000157086	0.012583627
GO_CHROMOSOME_ORGANIZATION	1028	46	1.86	0.000160308	0.012706486
GO_PROTEASOME_ASSEMBLY	12	4	19.28	0.000174087	0.013654955
GO_CELL_CYCLE_PROCESS	1227	52	1.76	0.000230038	0.017857599
GO_NEGATIVE_REGULATION_OF_CELL_CYCLE_PHASE_TRANSITION	226	16	2.97	0.000235303	0.018079919
GO_ESTABLISHMENT_OF_PROTEIN_LOCALIZATION_TO_CHROMOSOME	23	5	10.72	0.000240513	0.018293544
GO_REGULATION_OF_CELL_CYCLE_PROCESS	697	34	2.02	0.000248069	0.018679566
GO_RESPIRATORY_CHAIN_COMPLEX_IV_ASSEMBLY	24	5	10.16	0.000297489	0.022179124
GO_CELLULAR_RESPONSE_TO_STEROID_HORMONE_STIMULUS	232	16	2.89	0.000315801	0.023087217
GO_COFACTOR_BIOSYNTHETIC_PROCESS	210	15	3.00	0.000331893	0.024030347
GO_POSTTRANSCRIPTIONAL_REGULATION_OF_GENE_EXPRESSION	541	28	2.14	0.000339529	0.024349083
GO_PROTEIN_MODIFICATION_BY_SMALL_PROTEIN_REMOVAL	258	17	2.75	0.000353726	0.025127913
GO_NEGATIVE_REGULATION_OF_CELL_CYCLE_PROCESS	308	19	2.57	0.000374219	0.026335243
GO_STEROID_HORMONE_MEDIATED_SIGNALING_PATHWAY	170	13	3.22	0.000426116	0.029437169
GO_ESTABLISHMENT_OF_PROTEIN_LOCALIZATION_TO_MITOCHONDRIAL_MEMBRANE	15	4	14.02	0.000451747	0.030924135
GO_PROTEIN_INSERTION_INTO_MEMBRANE	55	7	5.64	0.000467402	0.031707571
GO_REGULATION_OF_CHROMOSOME_ORGANIZATION	315	19	2.51	0.000493284	0.032871028
GO_ORGANIC_CYCLIC_COMPOUND_CATABOLIC_PROCESS	584	29	2.05	0.000523426	0.034573684
GO_CHROMATIN_ORGANIZATION_INVOLVED_IN_REGULATION_OF_TRANSCRIPTION	74	8	4.69	0.00057524	0.037665695
GO_CRISTAE_FORMATION	16	4	12.85	0.000590269	0.038316592
GO_DNA_CONFORMATION_CHANGE	223	15	2.81	0.000623651	0.039864213
GO_NEGATIVE_REGULATION_OF_MITOTIC_CELL_CYCLE	296	18	2.53	0.000624698	0.039864213
GO_RIBOSOMAL_LARGE_SUBUNIT_ASSEMBLY	28	5	8.39	0.000632507	0.040023345
GO_MODIFICATION_DEPENDENT_MACROMOLECULE_CATABOLIC_PROCESS	592	29	2.02	0.000649056	0.040728256
GO_REGULATION_OF_CELL_CYCLE_G2_M_PHASE_TRANSITION	201	14	2.91	0.000665297	0.041402388
GO_RNA_CATABOLIC_PROCESS	375	21	2.32	0.000675981	0.041722426
GO_PROTEIN_MODIFICATION_BY_SMALL_PROTEIN_CONJUGATION	828	37	1.84	0.000710102	0.043472119
GO_NEGATIVE_REGULATION_OF_TRANSCRIPTION_BY_RNA_POLYMERASE_II	799	36	1.86	0.000720679	0.04376379
GO_AMINO_ACID_ACTIVATION	29	5	8.04	0.00074844	0.04437599

GO_REGULATION_OF_TRANSCRIPTION_ELONGATION_FROM_RNA_POLYMERASE_II_PROMOTER	29	5	8.04	0.00074844	0.04437599
GO_DNA_CATABOLIC_PROCESS_ENDONUCLEOLYTIC	29	5	8.04	0.00074844	0.04437599
GO_AEROBIC_ELECTRON_TRANSPORT_CHAIN	17	4	11.86	0.000756448	0.04450042
GO_INTRACELLULAR_RECEPTOR_SIGNALING_PATHWAY	253	16	2.63	0.000811854	0.047026827
GO_MATURATION_OF_SSU_RRNA	44	6	6.10	0.000818129	0.047026827
GO_PROTEIN_INSERTION_INTO_MITOCHONDRIAL_MEMBRANE	44	6	6.10	0.000818129	0.047026827
GO_RRNA_MODIFICATION	30	5	7.72	0.000879526	0.049904987
GO_TRANSCRIPTION_BY_RNA_POLYMERASE_I	61	7	5.01	0.000881456	0.049904987

ERRα- target genes

KEGG pathways

Geneset	Size	Overlap	OddRatio	p-value	q-value
KEGG_FC_GAMMA_R_MEDIATED_PHAGOCYTOSIS	91	8	9.23	5.78E-06	0.000661644
KEGG_FC_EPSILON_RI_SIGNALING_PATHWAY	67	7	11.13	7.11E-06	0.000661644
KEGG_B_CELL_RECEPTOR_SIGNALING_PATHWAY	74	6	8.39	0.00013737	0.008516963
KEGG_ENDOMETRIAL_CANCER	52	5	10.08	0.00022514	0.010469
KEGG_MAPK_SIGNALING_PATHWAY	245	10	4.08	0.000310042	0.01146718
KEGG_AXON_GUIDANCE	127	7	5.56	0.000426283	0.01146718
KEGG_CHEMOKINE_SIGNALING_PATHWAY	167	8	4.80	0.000431561	0.01146718
KEGG_T_CELL_RECEPTOR_SIGNALING_PATHWAY	100	6	6.06	0.000702865	0.013388429
KEGG_ADHERENS_JUNCTION	67	5	7.64	0.000735212	0.013388429
KEGG_VEGF_SIGNALING_PATHWAY	68	5	7.52	0.000786769	0.013388429
KEGG_PANCREATIC_CANCER	69	5	7.40	0.000840977	0.013388429
KEGG_NATURAL_KILLER_CELL_MEDIATED_CYTOTOXICITY	104	6	5.81	0.00086377	0.013388429
KEGG_PHOSPHATIDYLINOSITOL_SIGNALING_SYSTEM	75	5	6.76	0.001226664	0.017550738
KEGG_REGULATION_OF_ACTIN_CYTOSKELETON	201	8	3.95	0.001439074	0.019119123
KEGG_MTOR_SIGNALING_PATHWAY	48	4	8.58	0.00167812	0.020808685
KEGG_LYSOSOME	120	6	4.99	0.001806948	0.021005772
KEGG_INSULIN_SIGNALING_PATHWAY	128	6	4.66	0.002501488	0.026818779
KEGG_NON_SMALL_CELL_LUNG_CANCER	54	4	7.55	0.002595366	0.026818779
KEGG_PATHOGENIC_ESCHERICHIA_COLI_INFECTION	55	4	7.40	0.002775892	0.027174522
KEGG_COLORECTAL_CANCER	62	4	6.51	0.004285569	0.03985579
KEGG_LEISHMANIA_INFECTION	64	4	6.29	0.004801433	0.040846176
KEGG_FOCAL_ADHESION	194	7	3.55	0.004831268	0.040846176

GO BP

Geneset	Size	Overlap	OddRatio	p-value	q-value
GO_REGULATION_OF_ORGANELLE_ORGANIZATION	1164	34	3.08	9.41E-08	0.000708346
GO_CYTOSKELETON_ORGANIZATION	1212	32	2.74	2.08E-06	0.007815825
GO_POSITIVE_REGULATION_OF_CELLULAR_COMPONENT_ORGANIZATION	1103	29	2.71	7.04E-06	0.014679677
GO_POSITIVE_REGULATION_OF_ORGANELLE_ORGANIZATION	573	19	3.37	1.34E-05	0.019592739
GO_COAGULATION	291	13	4.53	1.56E-05	0.019592739
GO_PLATELET_ACTIVATION	143	9	6.44	2.32E-05	0.024943693
GO_MITOTIC_SISTER_CHROMATID_COHESION	18	4	26.98	3.39E-05	0.031887365
GO_MRNA_PROCESSING	465	16	3.47	4.20E-05	0.034512836
GO_POSITIVE_REGULATION_OF_CATALYTIC_ACTIVITY	1289	30	2.38	4.86E-05	0.034512836
GO_RESPONSE_TO_ENDOGENOUS_STIMULUS	1495	33	2.26	5.60E-05	0.034512836
GO_PEPTIDYL_AMINO_ACID_MODIFICATION	1113	27	2.47	5.96E-05	0.034512836
GO_REGULATION_OF_BODY_FLUID_LEVELS	432	15	3.49	6.59E-05	0.035443813
GO_RESPONSE_TO_PEPTIDE_HORMONE	386	14	3.65	7.28E-05	0.036528902
GO_REGULATION_OF_INTRACELLULAR_PROTEIN_TRANSPORT	207	10	4.88	7.88E-05	0.037088192

GO_MRNA_METABOLIC_PROCESS	779	21	2.72	9.71E-05	0.043021029
GO_REGULATION_OF_PROTEIN_TARGETING	72	6	8.64	0.000117923	0.049331273

Supplementary Table 2. Gene sets enriched within the up- and down-regulated genes by XCT-790 treatment.

Up-regulated genes with treatment

HALLMARK

Geneset	Size	Overlap	OddRatio	p-value	q-value
HALLMARK_UNFOLDED_PROTEIN_RESPONSE	107	28	4.56	1.98E-09	9.92E-08
HALLMARK_HYPOXIA	172	34	3.17	8.90E-08	2.22E-06
HALLMARK_TNFA_SIGNALING_VIA_NFKB	175	33	2.99	4.31E-07	7.19E-06
HALLMARK_P53_PATHWAY	179	29	2.48	4.38E-05	0.00054802
HALLMARK_APOPTOSIS	145	24	2.54	0.00013794	0.00137936
HALLMARK_MTORC1_SIGNALING	194	29	2.25	0.00018947	0.00157891
HALLMARK_HEME_METABOLISM	192	26	2.00	0.00176457	0.01260406
HALLMARK_CHOLESTEROL_HOMEOSTASIS	68	12	2.72	0.00360917	0.02255732

GO BP

Geneset	Size	Overlap	OddRatio	p-value	q-value
GO_RESPONSE_TO_ENDOPLASMIC_RETICULUM_STRESS	260	55	3.50	5.73E-13	4.29E-09
GO_ENDOPLASMIC_RETICULUM_UNFOLDED_PROTEIN_RESPONSE	115	33	5.20	4.71E-12	1.76E-08
GO_INTRINSIC_APOPTOTIC_SIGNALING_PATHWAY_IN_RESPONSE_TO_ENDOPLASMIC_RETICULUM_STRESS	53	20	7.76	3.40E-10	8.47E-07
GO_CELLULAR_RESPONSE_TO_TOPOLOGICALLY_INCORRECT_PROTEIN	149	35	3.96	4.92E-10	9.20E-07
GO_RESPONSE_TO_TOPOLOGICALLY_INCORRECT_PROTEIN	183	39	3.50	1.08E-09	1.62E-06
GO_PROCESS_UTILIZING_AUTOPHAGIC_MECHANISM	467	69	2.26	1.64E-08	2.05E-05
GO_POSITIVE_REGULATION_OF_TRANSCRIPTION_BY_RNA_POLYMERASE_II	935	112	1.79	1.27E-07	0.00013519
GO_IRE1_MEDIATED_UNFOLDED_PROTEIN_RESPONSE	61	18	5.35	2.10E-07	0.00018894
GO_CIRCULATORY_SYSTEM_DEVELOPMENT	775	96	1.85	2.27E-07	0.00018894
GO_INTEGRATED_STRESS_RESPONSE_SIGNALING	25	11	10.01	5.39E-07	0.00037593
GO_T_CELL_PROLIFERATION	134	27	3.24	1.25E-06	0.00077868
GO_RESPONSE_TO_STARVATION	160	30	2.96	1.61E-06	0.00092821
GO_REGULATION_OF_AUTOPHAGY	286	44	2.34	2.20E-06	0.00117794
GO_MACROAUTOPHAGY	282	43	2.32	3.63E-06	0.00172507
GO_SELECTIVE_AUTOPHAGY	53	15	5.04	3.85E-06	0.00172507
GO_ER_NUCLEUS_SIGNALING_PATHWAY	47	14	5.41	4.15E-06	0.00172507
GO_INTRACILIARY_TRANSPORT	47	14	5.41	4.15E-06	0.00172507
GO_RESPONSE_TO_EXTRACELLULAR_STIMULUS	354	50	2.12	5.73E-06	0.00225478

GO_INTRACILIARY_TRANSPORT_INVOLVED_IN_CILIUM_ASSEMBLY	37	12	6.11	7.58E-06	0.00283607
GO_CELLULAR_RESPONSE_TO_GLUCOSE_STARVATION	44	13	5.34	1.01E-05	0.00359581
GO_TUBE_DEVELOPMENT	737	86	1.72	1.08E-05	0.00367419
GO_POSITIVE_REGULATION_OF_TRANSCRIPTION_FROM_RNA_POLYMERASE_II_PROMOTER_IN_RESPONSE_TO_STRESS	22	9	8.80	1.23E-05	0.00396754
GO_CELLULAR_RESPONSE_TO_STARVATION	134	25	2.94	1.27E-05	0.00396754
GO_REGULATION_OF_RESPONSE_TO_ENDOPLASMIC_RETICULUM_STRESS	73	17	3.87	1.59E-05	0.00451933
GO_POSITIVE_REGULATION_OF_NUCLEOBASE_CONTAINING_COMPOUND_METABOLIC_PROCESS	1496	152	1.49	1.63E-05	0.00451933
GO_ALPHA_BETA_T_CELL_PROLIFERATION	28	10	7.07	1.67E-05	0.00451933
GO_REGULATION_OF_CELL_CELL_ADHESION	290	42	2.18	1.71E-05	0.00451933
GO_LEUKOCYTE_CELL_CELL_ADHESION	243	37	2.31	1.75E-05	0.00451933
GO_VACUOLE_ORGANIZATION	154	27	2.72	1.90E-05	0.00473724
GO_GRANULOCYTE_MACROPHAGE_COLONY_STIMULATING_FACTOR_PRODUCTION	14	7	12.70	2.43E-05	0.00565696
GO_PROTEIN_TRANSPORT_ALONG_MICROTUBULE	61	15	4.16	2.49E-05	0.00565696
GO_POSITIVE_REGULATION_OF_TRANSCRIPTION_FROM_RNA_POLYMERASE_II_PROMOTER_IN_RESPONSE_TO_ENDOPLASMIC_RETICULUM_STRESS	10	6	19.02	2.50E-05	0.00565696
GO_POSITIVE_REGULATION_OF_CELL_ADHESION	295	42	2.14	2.59E-05	0.00569339
GO_HEART_DEVELOPMENT	406	53	1.94	2.93E-05	0.00625817
GO_REGULATION_OF_T_CELL_ACTIVATION	223	34	2.31	3.68E-05	0.00764311
GO_APOPTOTIC_SIGNALING_PATHWAY	473	59	1.84	4.08E-05	0.00803837
GO_EPITHELIUM_DEVELOPMENT	837	92	1.60	5.48E-05	0.01051393
GO_TUBE_MORPHOGENESIS	596	70	1.72	5.80E-05	0.01063116
GO_INTRINSIC_APOPTOTIC_SIGNALING_PATHWAY	247	36	2.19	5.83E-05	0.01063116
GO_VASCULATURE_DEVELOPMENT	512	62	1.78	6.33E-05	0.01128143
GO_SPECIFICATION_OF_SYMMETRY	89	18	3.24	6.81E-05	0.01183959
GO_REGULATION_OF_ALPHA_BETA_T_CELL_ACTIVATION	67	15	3.68	8.05E-05	0.01368656
GO_SERINE_FAMILY_AMINO_ACID_METABOLIC_PROCESS	33	10	5.53	8.42E-05	0.01400474
GO_RESPONSE_TO_LAMINAR_FLUID_SHEAR_STRESS	12	6	12.69	9.66E-05	0.015546
GO_POSITIVE_REGULATION_OF_DEVELOPMENTAL_PROCESS	889	95	1.55	0.00011866	0.01849308
GO_MITOPHAGY	23	8	6.77	0.00014795	0.02128762
GO_INTERLEUKIN_17_PRODUCTION	23	8	6.77	0.00014795	0.02128762
GO_PATTERN_SPECIFICATION_PROCESS	288	39	2.01	0.00014797	0.02128762
GO_CILIUM_ORGANIZATION	339	44	1.92	0.00015441	0.02179564
GO_REGULATION_OF_MULTICELLULAR_ORGANISMAL_DEVELOPMENT	943	99	1.52	0.00016514	0.02217838
GO_GOLGI_ORGANIZATION	129	22	2.63	0.00016547	0.02217838
GO_VENTRICULAR_SEPTUM_DEVELOPMENT	49	12	4.13	0.00016602	0.02217838
GO_POSITIVE_REGULATION_OF_PROTEIN_METABOLIC_PROCESS	1200	121	1.46	0.00017397	0.02283235
GO_GOLGI_VESICLE_TRANSPORT	342	44	1.90	0.00018852	0.02431615
GO_ORGANONITROGEN_COMPOUND_CATABOLIC_PROCESS	1098	112	1.48	0.00019541	0.02443412
GO_EPITHELIAL_CELL_DIFFERENTIATION	447	54	1.77	0.00019597	0.02443412

GO_REGULATION_OF_AUTOPHAGY_OF_MITOCHONDRION	30	9	5.45	0.00020783	0.02548806
GO_AUTOPHAGOSOME_ORGANIZATION	89	17	3.01	0.00022304	0.0269118
GO_POSITIVE_REGULATION_OF_TRANSCRIPTION_FROM_RNA_POLYMERASE_II_PROMOTER_INVOLVED_IN_CELLULAR_RESPONSE_TO_CHEMICAL_STIMULUS	19	7	7.41	0.00025761	0.03059047
GO_NEUROGENESIS	1201	120	1.45	0.00026751	0.03126966
GO_PROTEIN_DEGLYCOSYLATION	25	8	5.98	0.00028597	0.0324144
GO_REGULATION_OF_ENDOPLASMIC_RETICULUM_UNFOLDED_PROTEIN_RESPONSE	25	8	5.98	0.00028597	0.0324144
GO_POSITIVE_REGULATION_OF_CELL_DIFFERENTIATION	596	67	1.63	0.00029807	0.03328138
GO_REGULATION_OF_MACROAUTOPHAGY	144	23	2.43	0.00032202	0.03542711
GO_REGULATION_OF_APOPTOTIC_SIGNALING_PATHWAY	279	37	1.96	0.00032807	0.03556975
GO_CELLULAR_RESPONSE_TO_EXTRACELLULAR_STIMULUS	191	28	2.20	0.00033666	0.03597892
GO_INTRA_GOLGI_VESICLE_MEDIATED_TRANSPORT	32	9	4.97	0.00035657	0.03673685
GO_POSITIVE_REGULATION_OF_RESPONSE_TO_ENDOPLASMIC_RETICULUM_STRESS	32	9	4.97	0.00035657	0.03673685
GO_REGULATION_OF_PROGRAMMED_NECROTIC_CELL_DEATH	20	7	6.84	0.00037136	0.03673685
GO_DORSAL_VENTRAL_PATTERN_FORMATION	46	11	4.00	0.00038561	0.03673685
GO_VENTRAL_SPINAL_CORD_DEVELOPMENT	26	8	5.64	0.00038671	0.03673685
GO_RESPONSE_TO_FLUID_SHEAR_STRESS	26	8	5.64	0.00038671	0.03673685
GO_REGULATION_OF_ENDOPLASMIC_RETICULUM_STRESS_INDUCED_INTRINSIC_APOPTOTIC_SIGNALING_PATHWAY	26	8	5.64	0.00038671	0.03673685
GO_L_SERINE_METABOLIC_PROCESS	10	5	12.68	0.00038794	0.03673685
GO_NEGATIVE_REGULATION_OF_AUTOPHAGY	69	14	3.24	0.00041099	0.03843261
GO_SERINE_FAMILY_AMINO_ACID_BIOSYNTHETIC_PROCESS	15	6	8.46	0.00043172	0.03938701
GO_REGULATION_OF_TRANSLATIONAL_INITIATION_IN_RESPONSE_TO_STRESS	15	6	8.46	0.00043172	0.03938701
GO_LEUKOCYTE_PROLIFERATION	204	29	2.12	0.00044868	0.04044053
GO_REGULATION_OF_LEUKOCYTE_PROLIFERATION	158	24	2.29	0.00051592	0.04594224
GO_REGULATION_OF_TRANSLATION_IN_RESPONSE_TO_STRESS	21	7	6.35	0.000522	0.04594224
GO_POSITIVE_REGULATION_OF_CELL_CELL_ADHESION	187	27	2.16	0.0005433	0.04726042

Down-regulated genes with treatment

HALLMARK

Geneset	Size	Overlap	OddRatio	p-value	q-value
HALLMARK_MYC_TARGETS_V2	57	36	24.11	3.32E-28	1.66E-26
HALLMARK_OXIDATIVE_PHOSPHORYLATION	182	60	7.03	6.45E-26	1.61E-24
HALLMARK_MYC_TARGETS_V1	193	57	5.97	5.24E-22	8.73E-21
HALLMARK_E2F_TARGETS	193	42	3.90	1.27E-11	1.59E-10
HALLMARK_DNA_REPAIR	144	31	3.82	8.03E-09	8.03E-08
HALLMARK_MTORC1_SIGNALING	194	37	3.29	9.88E-09	8.23E-08
HALLMARK_UV_RESPONSE_UP	133	27	3.54	2.60E-07	1.86E-06
HALLMARK_G2M_CHECKPOINT	190	31	2.71	5.03E-06	3.14E-05
HALLMARK_ESTROGEN_RESPONSE_EARLY	165	21	2.01	0.00421496	0.02341645

KEGG

Geneset	Size	Overlap	OddRatio	p-value	q-value
KEGG_PARKINSONS_DISEASE	90	34	8.51	2.43E-17	2.33E-15
KEGG_SPLICEOSOME	124	40	6.70	2.50E-17	2.33E-15
KEGG_OXIDATIVE_PHOSPHORYLATION	93	33	7.70	6.35E-16	3.94E-14
KEGG_HUNTINGTONS_DISEASE	147	41	5.44	3.08E-15	1.43E-13
KEGG_ALZHEIMERS_DISEASE	133	32	4.42	2.34E-10	8.71E-09
KEGG_PYRIMIDINE_METABOLISM	92	25	5.18	1.43E-09	4.44E-08
KEGG_RNA_POLYMERASE	28	12	10.34	1.05E-07	2.79E-06
KEGG_PURINE_METABOLISM	133	27	3.54	2.60E-07	6.04E-06
KEGG_BASE_EXCISION_REPAIR	33	9	5.15	0.0002683	0.00554489
KEGG_CARDIAC_MUSCLE_CONTRACTION	50	10	3.43	0.00175708	0.03268176
KEGG_DNA_REPLICATION	35	8	4.07	0.00205493	0.03474694
KEGG_RNA_DEGRADATION	54	10	3.12	0.00319642	0.04954449

GO BP

Geneset	Size	Overlap	OddRatio	p-value	q-value
GOBP_RNA_PROCESSING	1019	213	4.19	6.87E-53	5.14E-49
GOBP_RIBONUCLEOPROTEIN_COMPLEX_BIOGENESIS	420	130	6.77	8.19E-52	3.06E-48
GOBP_RIBOSOME_BIOGENESIS	290	106	8.56	4.40E-50	1.10E-46
GOBP_NCRNA_PROCESSING	367	111	6.44	3.99E-43	7.46E-40
GOBP_NCRNA_METABOLIC_PROCESS	433	119	5.65	1.68E-41	2.51E-38
GOBP_RRNA_METABOLIC_PROCESS	225	82	8.35	9.59E-39	1.20E-35
GOBP_MITOCHONDRIAL_GENE_EXPRESSION	158	60	8.76	9.28E-30	9.92E-27
GOBP_MITOCHONDRION_ORGANIZATION	469	103	4.12	4.10E-27	3.84E-24
GOBP_MRNA_PROCESSING	476	101	3.93	2.34E-25	1.94E-22
GOBP_RNA_SPLICING	421	93	4.12	9.53E-25	7.13E-22
GOBP_MRNA_METABOLIC_PROCESS	783	135	3.09	2.25E-24	1.53E-21
GOBP_RNA_SPLICING_VIA_TRANSESTERIFICATION_REACTION S	335	80	4.53	1.04E-23	6.45E-21
GOBP_MITOCHONDRIAL_TRANSLATION	130	47	8.01	1.65E-22	9.50E-20
GOBP_MITOCHONDRIAL_RESPIRATORY_CHAIN_COMPLEX_AS SEMBLY	91	38	10.09	4.90E-21	2.62E-18
GOBP_MITOCHONDRIAL_TRANSLATIONAL_TERMINATION	88	37	10.20	1.21E-20	6.05E-18
GOBP_TRANSLATIONAL_TERMINATION	103	39	8.57	9.85E-20	4.61E-17
GOBP_TRANSLATIONAL_ELONGATION	127	43	7.22	1.95E-19	8.56E-17
GOBP_OXIDATIVE_PHOSPHORYLATION	106	38	7.85	2.63E-18	1.09E-15
GOBP_RESPIRATORY_ELECTRON_TRANSPORT_CHAIN	97	36	8.28	5.52E-18	2.17E-15
GOBP_ATP_SYNTHESIS_COUPLED_ELECTRON_TRANSPORT	82	33	9.44	7.43E-18	2.78E-15

GOBP_ELECTRON_TRANSPORT_CHAIN	144	42	5.79	2.43E-16	8.25E-14
GOBP_PEPTIDE_BIOSYNTHETIC_PROCESS	583	95	2.81	1.03E-15	3.35E-13
GOBP_CELLULAR_RESPIRATION	157	43	5.31	1.34E-15	4.17E-13
GOBP_ATP_METABOLIC_PROCESS	245	54	4.00	8.39E-15	2.51E-12
GOBP_RIBOSOMAL_LARGE_SUBUNIT_BIOGENESIS	69	27	8.96	1.70E-14	4.88E-12
GOBP_CELLULAR_PROTEIN_CONTAINING_COMPLEX_ASSEMBLY	867	120	2.33	3.95E-14	1.10E-11
GOBP_NADH_DEHYDROGENASE_COMPLEX_ASSEMBLY	56	24	10.43	4.32E-14	1.15E-11
GOBP_NUCLEAR_TRANSPORT	311	60	3.39	1.52E-13	3.91E-11
GOBP_GENERATION_OF_PRECURSOR_METABOLITES_AND_ENERGY	417	72	2.97	1.91E-13	4.77E-11
GOBP_AMIDE_BIOSYNTHETIC_PROCESS	698	101	2.44	3.13E-13	7.55E-11
GOBP_CELLULAR_PROTEIN_COMPLEX_DISASSEMBLY	198	45	4.14	4.68E-13	1.09E-10
GOBP_RIBOSOMAL_SMALL_SUBUNIT_BIOGENESIS	72	26	7.87	4.81E-13	1.09E-10
GOBP_ENERGY_DERIVATION_BY_OXIDATION_OF_ORGANIC_COMPOUNDS	228	48	3.76	1.58E-12	3.48E-10
GOBP_MITOCHONDRIAL_RNA_METABOLIC_PROCESS	43	19	10.97	1.00E-11	2.15E-09
GOBP_PEPTIDE_METABOLIC_PROCESS	707	97	2.28	2.19E-11	4.56E-09
GOBP_RNA_EXPORT_FROM_NUCLEUS	131	33	4.70	3.27E-11	6.62E-09
GOBP_TRNA_PROCESSING	121	31	4.80	8.10E-11	1.59E-08
GOBP_RIBONUCLEOPROTEIN_COMPLEX_SUBUNIT_ORGANIZATION	191	40	3.71	1.36E-10	2.54E-08
GOBP_CYTOCHROME_COMPLEX_ASSEMBLY	34	16	12.29	1.36E-10	2.54E-08
GOBP_PROTEIN_CONTAINING_COMPLEX_DISASSEMBLY	293	52	3.04	1.63E-10	2.98E-08
GOBP_RIBOSOME_ASSEMBLY	60	21	7.47	1.67E-10	2.98E-08
GOBP_POSTTRANSCRIPTIONAL_REGULATION_OF_GENE_EXPRESSION	541	78	2.39	2.13E-10	3.70E-08
GOBP_PROTEIN_TARGETING_TO_MITOCHONDRION	93	26	5.40	3.41E-10	5.80E-08
GOBP_CELLULAR_AMIDE_METABOLIC_PROCESS	933	114	2.00	5.47E-10	9.09E-08
GOBP_TRNA_METABOLIC_PROCESS	153	34	3.99	6.32E-10	1.03E-07
GOBP_RNA_LOCALIZATION	218	42	3.34	7.26E-10	1.16E-07
GOBP_MATURATION_OF_SSU_RRNA	48	18	8.30	9.24E-10	1.44E-07
GOBP_MITOCHONDRIAL_TRANSPORT	229	43	3.24	1.05E-09	1.60E-07
GOBP_NUCLEAR_EXPORT	189	38	3.52	1.33E-09	2.00E-07
GOBP_PROTEIN_LOCALIZATION_TO_MITOCHONDRION	135	31	4.15	1.53E-09	2.25E-07
GOBP_MITOCHONDRIAL_ELECTRON_TRANSPORT_NADH_TO_UBIQUINONE	46	17	8.11	3.53E-09	5.07E-07
GOBP_REGULATION_OF_RNA_SPLICING	132	30	4.10	3.64E-09	5.14E-07
GOBP_MATURATION_OF_LSU_RRNA_FROM_TRICISTRONIC_RRNA_TRANSCRIPT_SSU_RRNA_5_8S_RRNA_LSU_RRNA	15	10	27.53	4.61E-09	6.38E-07
GOBP_NUCLEOBASE_CONTAINING_COMPOUND_TRANSPORT	225	41	3.12	6.40E-09	8.70E-07
GOBP_MRNA_EXPORT_FROM_NUCLEUS	106	26	4.52	7.28E-09	9.73E-07
GOBP_ESTABLISHMENT_OF_RNA_LOCALIZATION	185	36	3.37	8.99E-09	1.18E-06
GOBP_MATURATION_OF_LSU_RRNA	28	13	11.95	9.26E-09	1.19E-06
GOBP_REGULATION_OF_MRNA_METABOLIC_PROCESS	300	49	2.74	9.62E-09	1.22E-06
GOBP_RESPIRATORY_CHAIN_COMPLEX_IV_ASSEMBLY	24	12	13.78	1.21E-08	1.50E-06

GOBP_DNA_TEMPLATED_TRANSCRIPTION_TERMINATION	69	20	5.65	1.88E-08	2.30E-06
GOBP_TRANSCRIPTION_BY_RNA_POLYMERASE_I	58	18	6.23	2.96E-08	3.57E-06
GOBP_RNA_5_END_PROCESSING	22	11	13.77	4.98E-08	5.92E-06
GOBP_RNA_MODIFICATION	150	30	3.48	8.10E-08	9.47E-06
GOBP_MATURATION_OF_5_8S_RRNA	34	13	8.54	1.56E-07	1.79E-05
GOBP_RNA_PHOSPHODIESTER_BOND_HYDROLYSIS	138	28	3.54	1.59E-07	1.80E-05
GOBP_MATURATION_OF_SSU_RRNA_FROM_TRICISTRONIC_RRNA_TRANSCRIPT_SSU_RRNA_5_8S_RRNA_LSU_RRNA	35	13	8.15	2.33E-07	2.60E-05
GOBP_MRNA_TRANSPORT	141	28	3.44	2.54E-07	2.79E-05
GOBP_MITOCHONDRIAL_CYTOCHROME_C_OXIDASE_ASSEMBLY	21	10	12.51	3.70E-07	4.01E-05
GOBP_PROTEIN_CONTAINING_COMPLEX_LOCALIZATION	250	40	2.66	3.81E-07	4.07E-05
GOBP_RNA_3_END_PROCESSING	136	27	3.44	4.16E-07	4.39E-05
GOBP_DNA_TEMPLATED_TRANSCRIPTION_INITIATION	208	35	2.82	5.99E-07	6.16E-05
GOBP_PROTEIN_FOLDING	199	34	2.87	6.01E-07	6.16E-05
GOBP_REGULATION_OF_TRANSCRIPTION_BY_RNA_POLYMERASE_I	28	11	8.91	1.04E-06	0.00010332
GOBP_NEGATIVE_REGULATION_OF_GENE_EXPRESSION	932	102	1.74	1.13E-06	0.0001116
GOBP_CELLULAR_COMPONENT_DISASSEMBLY	475	61	2.07	1.27E-06	0.00012354
GOBP_MATURATION_OF_5_8S_RRNA_FROM_TRICISTRONIC_RRNA_TRANSCRIPT_SSU_RRNA_5_8S_RRNA_LSU_RRNA	24	10	9.83	1.70E-06	0.00016343
GOBP_MITOCHONDRIAL_TRANSCRIPTION	15	8	15.70	1.91E-06	0.00018103
GOBP_NUCLEIC_ACID_PHOSPHODIESTER_BOND_HYDROLYSIS	266	40	2.47	1.94E-06	0.00018103
GOBP_RNA_CATABOLIC_PROCESS	377	51	2.19	2.09E-06	0.00019281
GOBP_NCRNA_TRANSCRIPTION	99	21	3.73	2.60E-06	0.0002373
GOBP_ORGANIC_CYCLIC_COMPOUND_CATABOLIC_PROCESS	541	66	1.95	2.85E-06	0.0002573
GOBP_MITOCHONDRIAL_MEMBRANE_ORGANIZATION	109	22	3.50	3.62E-06	0.00032204
GOBP_MITOCHONDRIAL_ELECTRON_TRANSPORT_UBIQUINOL_TO_CYTOCHROME_C	12	7	19.22	3.95E-06	0.00034746
GOBP_MRNA_3_END_PROCESSING	94	20	3.74	4.26E-06	0.0003708
GOBP_ORGANONITROGEN_COMPOUND_BIOSYNTHETIC_PROCESS	1400	138	1.56	5.05E-06	0.00043393
GOBP_REGULATION_OF_GENE_SILENCING_BY_RNA	88	19	3.81	5.87E-06	0.00049873
GOBP_REGULATION_OF_MITOCHONDRIAL_GENE_EXPRESSION	27	10	8.09	6.07E-06	0.00051004
GOBP_SPLICEOSOMAL_SNRNP_ASSEMBLY	39	12	6.12	6.68E-06	0.00055567
GOBP_REGULATION_OF_MRNA_SPLICING_VIA_SPLICEOSOME	90	19	3.70	8.30E-06	0.00068227
GOBP_REGULATION_OF_VIRAL_TRANSCRIPTION	40	12	5.90	8.96E-06	0.00072808
GOBP_REGULATION_OF_CELLULAR_RESPONSE_TO_HEAT	75	17	4.05	9.05E-06	0.00072808
GOBP_TRNA_MODIFICATION	83	18	3.83	9.61E-06	0.00075739
GOBP_CELL_CYCLE_G1_S_PHASE_TRANSITION	244	36	2.41	9.62E-06	0.00075739
GOBP_REGULATION_OF_MRNA_PROCESSING	128	23	3.03	1.67E-05	0.00129808
GOBP_PROTEIN_IMPORT_INTO_MITOCHONDRIAL_MATRIX	19	8	9.99	1.76E-05	0.00135411
GOBP_CHAPERONE_MEDIATED_PROTEIN_FOLDING	57	14	4.49	2.10E-05	0.00160404
GOBP_POSITIVE_REGULATION_OF_TYPE_I_INTERFERON_PRODUCTION	65	15	4.14	2.40E-05	0.00181456
GOBP_DNA_TEMPLATED_TRANSCRIPTION_ELONGATION	105	20	3.25	2.43E-05	0.00181585

GOBP_REGULATION_OF_GENE_SILENCING	97	19	3.37	2.56E-05	0.0018686
GOBP_RNA_PHOSPHODIESTER_BOND_HYDROLYSIS_ENDONUCLEOLYTIC	73	16	3.87	2.58E-05	0.0018686
GOBP_PROTEIN_TRANSMEMBRANE_TRANSPORT	58	14	4.39	2.60E-05	0.0018686
GOBP_POSITIVE_REGULATION_OF_MITOCHONDRIAL_TRANSLATION	15	7	12.01	2.67E-05	0.0018862
GOBP_TRNA_5_END_PROCESSING	15	7	12.01	2.67E-05	0.0018862
GOBP_MITOTIC_CELL_CYCLE	907	94	1.63	2.76E-05	0.00193316
GOBP_POSITIVE_REGULATION_OF_VIRAL_TRANSCRIPTION	26	9	7.28	3.36E-05	0.00230333
GOBP_TRANSCRIPTION_BY_RNA_POLYMERASE_III	45	12	5.01	3.36E-05	0.00230333
GOBP_DNA_REPLICATION	260	36	2.23	3.91E-05	0.00265689
GOBP_TERMINATION_OF_RNA_POLYMERASE_I_TRANSCRIPTION	27	9	6.87	4.73E-05	0.00318778
GOBP_REGULATION_OF_ATPASE_ACTIVITY	69	15	3.83	5.07E-05	0.00338949
GOBP_TRANSCRIPTION_INITIATION_FROM_RNA_POLYMERASE_II_PROMOTER	166	26	2.57	5.81E-05	0.00384783
GOBP_RRNA_MODIFICATION	34	10	5.73	6.08E-05	0.00398813
GOBP_TRNA_5_LEADER_REMOVAL	12	6	13.72	6.43E-05	0.00418419
GOBP_REGULATION_OF_MRNA_CATABOLIC_PROCESS	187	28	2.44	6.98E-05	0.00450019
GOBP_INTRACELLULAR_PROTEIN_TRANSPORT	1040	103	1.55	7.19E-05	0.00459712
GOBP_POSITIVE_REGULATION_OF_RNA_SPLICING	35	10	5.50	7.99E-05	0.00506667
GOBP_MITOCHONDRIAL_TRANSMEMBRANE_TRANSPORT	80	16	3.45	8.41E-05	0.00528415
GOBP_AEROBIC_RESPIRATION	72	15	3.63	8.53E-05	0.00531739
GOBP_PROTEIN_IMPORT	170	26	2.50	8.74E-05	0.00540216
GOBP_CELLULAR_RESPONSE_TO_HEAT	106	19	3.02	9.12E-05	0.00559403
GOBP_RNA_METHYLATION	73	15	3.56	0.00010068	0.00603658
GOBP_REGULATION_OF_CELLULAR_AMIDE_METABOLIC_PROCESS	368	45	1.94	0.00010079	0.00603658
GOBP_CELL_CYCLE_PROCESS	1198	115	1.50	0.00010087	0.00603658
GOBP_PSEUDOURIDINE_SYNTHESIS	18	7	8.73	0.00011019	0.00654235
GOBP_DNA_DEPENDENT_DNA_REPLICATION	145	23	2.61	0.00012509	0.00736865
GOBP_RESPONSE_TO_HEAT	136	22	2.67	0.00012978	0.00758511
GOBP_CHAPERONE_COFACTOR_DEPENDENT_PROTEIN_REFOLDING	31	9	5.62	0.00015873	0.00913451
GOBP_DNA_STRAND_ELONGATION_INVOLVED_IN_DNA_REPLICATION	19	7	8.01	0.00016423	0.00937846
GOBP_PROTEIN_INSERTION_INTO_MEMBRANE	68	14	3.57	0.00016638	0.00942948
GOBP_IMPORT_INTO_NUCLEUS	139	22	2.60	0.00017968	0.01010641
GOBP_MITOCHONDRIAL_ELECTRON_TRANSPORT_CYTOCHROME_C_TO_OXYGEN	14	6	10.29	0.00018561	0.01036224
GOBP_DE_NOVO_PROTEIN_FOLDING	39	10	4.74	0.00021552	0.01194326
GOBP_RIBOSOMAL_LARGE_SUBUNIT_ASSEMBLY	26	8	6.10	0.00023609	0.01298669
GOBP_SNRNA_TRANSCRIPTION	71	14	3.38	0.00026834	0.01465305
GOBP_RRNA_PSEUDOURIDINE_SYNTHESIS	10	5	13.71	0.00027636	0.01498152
GOBP_REGULATION_OF_CELL_CYCLE	1029	99	1.50	0.00028634	0.01541065
GOBP_POSITIVE_REGULATION_OF_ATPASE_ACTIVITY	48	11	4.09	0.00030808	0.01634586
GOBP_CLEAVAGE_INVOLVED_IN_RRNA_PROCESSING	27	8	5.78	0.00031558	0.01662578

GOBP_POSITIVE_REGULATION_OF_TRANSCRIPTION_BY_RNA_POLYMERASE_I	21	7	6.86	0.00033586	0.01757058
GOBP_TYPE_I_INTERFERON_PRODUCTION	108	18	2.76	0.00035433	0.01840808
GOBP_ESTABLISHMENT_OF_PROTEIN_LOCALIZATION_TO_MITOCHONDRIAL_MEMBRANE	49	11	3.98	0.00037309	0.01924872
GOBP_PROTEIN_TRANSMEMBRANE_IMPORT_INTO_INTRACELLULAR_ORGANELLE	35	9	4.76	0.00043408	0.02224222
GOBP_MITOCHONDRIAL_RNA_PROCESSING	16	6	8.23	0.00043962	0.02237281
GOBP_PROTEIN_REFOLDING	22	7	6.40	0.00046378	0.02344271
GOBP_CHAPERONE_MEDIATED_PROTEIN_TRANSPORT	11	5	11.42	0.00047821	0.02401024
GOBP_APOPTOTIC_MITOCHONDRIAL_CHANGES	102	17	2.76	0.00051124	0.02549714
GOBP_POSITIVE_REGULATION_OF_MITOCHONDRION_ORGANIZATION	76	14	3.11	0.00055704	0.0275974
GOBP_BIOLOGICAL_PROCESS_INVOLVED_IN_SYMBIOTIC_INTERACTION	850	83	1.51	0.00056257	0.02768789
GOBP_MRNA_MODIFICATION	23	7	6.00	0.00062772	0.0306925
GOBP_POSITIVE_REGULATION_OF_MRNA_METABOLIC_PROCESS	78	14	3.01	0.00073059	0.0352615
GOBP_REGULATION_OF_GENERATION_OF_PRECURSOR_METABOLITES_AND_ENERGY	135	20	2.40	0.00082793	0.03968617
GOBP_TRNA_METHYLATION	38	9	4.26	0.00083287	0.03968617
GOBP_CELL_CYCLE_DNA_REPLICATION	62	12	3.30	0.00085999	0.04071874
GOBP_TRNA_WOBBLE_BASE_MODIFICATION	18	6	6.86	0.00090549	0.0426036
GOBP_GENE_SILENCING	197	26	2.10	0.00091902	0.04296979
GOBP_TRANSCRIPTION_ELONGATION_FROM_RNA_POLYMERASE_II_PROMOTER	80	14	2.92	0.00094778	0.04403931
GOBP_CELL_CYCLE_PHASE_TRANSITION	560	58	1.61	0.00095555	0.04412637
GOBP_ESTABLISHMENT_OF_PROTEIN_LOCALIZATION_TO_ORGANELLE	515	54	1.63	0.00106728	0.04898351
GOBP_DNA_STRAND_ELONGATION	25	7	5.34	0.00109139	0.04963462
GOBP_REGULATION_OF_MITOCHONDRION_ORGANIZATION	138	20	2.34	0.00109473	0.04963462

Supplementary Table 3. Clinical information of AML patients participated in this study.

25 AML patients

**Sample**	**FAB Classification**	**Risk stratification**	**Cytogenetic characteristics**	**Molecular characteristics**	**Blast (%)**	**Treatment**	**Note**
#2	N/A	Poor	N/A	*FLT3-TKD, TET2, RUNX1, KRAS, SRSF2, BCOR, IKZF1, NF1, ARID2,*	90	Supportive	
#7	5	Poor	Complex karyotype	N/A	28.4	Supportive	
#8	2	Intermediate	Normal	N/A	20.9	Supportive	
#9	2	Intermediate	t(7;11)	*WT1, PTPN11, FBXW7, PIK3R1, EP300*	47	IA	
#11	2	Poor	Normal	*FLT3-TKD, NPM1, DNMT3A, TP53, NRAS, SMC3, PRKD2, UNC12D*	61.4	DV	
#14	2	Intermediate	t(8;21)(q22;q22)	*FLT3-ITD (43.54), KIT, SMC3, MGA*	68.9	FLAG	Relapse
#17	2	Intermediate	Normal	*NPM1, TET2, WT1, EZH2, ZBTB7A,*	60	IA(3+5)	Relapse

				KMT2C, NF1, CIITA, EED, INFGR2			
#22	4	Intermediate	46,XX,add(12)(p11)	*TET2, RCORL1*	25.5	DV	Relapse
#24	2	Poor	45,XY,add(12)(p11),-17	*DNMT3A, RUNX1, TP53, ASXL1, STAG2, BCOR, ATM, U2AF2, IL6R*	28.6	IV->FLAG	
#27	4	Intermediate	Normal	*FLT3-ITD (45.97), NPM1, DNMT3A, JAK1, ALK, BCL6, MAP2K1*	94.6	IA	
#28	2	Intermediate	Normal	*FLT3-TKD, WT1, KMT2A, BIRC3, HIST1H1E*	48.6	IA	
#29	4	Poor	47,XX,+11	*FLT3-ITD (76.47), WT1, KIT, U2AF1*	86.2	IA	
#31	1	Poor	46,XY,dup(7)(q22q36)	*FLT3-ITD (45.56), RUNX1, NKFBIE,*	95.7	IA	
#32	1	Poor	46,XY,dup(20)(q13.1)	*DNMT3A, RUNX1, PHF6, SRSF2, MGA*	83.6	IA	
#35	5	Intermediate	45,X,-Y,del(9)(q22)	*FLT3-TKD, NPM1, NRAS, PTPN11,*	57.4	DV	
#36	1	Intermediate	Normal	N/A	86.3	N/A	
#41	2	Favorable	Normal	*CEBPA, WT1, ARID1A, ARID2, CSF3R, NOTCH1, NOTCH2*	20.3	DV	
#53	1	Intermediate	47,XX,+6	N/A	88.5	Supportive	
#54	N/A	Favorable	Normal	*NPM1, DNMT3A, IDH2, PTPN11, ZFHX4, TP63*	N/A	IA	
#57	2	Intermediate	47,XX,+8	N/A	28.7	D	
#58	2	Poor	Complex karyotype	N/A	24	DV	
#59	N/A	Poor	Normal	*TET2, ASXL1, STAG2, SRSF2, NF1*	23.5	D	Secondary
#62	4	Favorable	Normal	*FLT3-ITD (26.1), CEBPA, RAD21, BCOR, IKZF1, KDM5A, RPS15, STAT5B*	89.4	DV	
#64	N/A	Favorable	45,X,-Y,t(8;21)	*KIT, SMC1A, BCR, ZBTB7A*	38.1	DV	
#71	5	Poor	Complex karyotype	*TP53*	95.6	IA	Persistent

IA: idarubicin + Ara-C
DV: decitabine + venetoclax
FLAG: fludarabine + high-dose cytarabine + G-CSF
D: decitabine

Supplementary Table 4. Information of reagents and antibodies used in this study.

**Reagents**	**Source**	**Cat# and Identifier**
**Antibodies**		
Rabbit polyclonal anti-caspase-3 antibody (1:1,000)	Cell Signaling Technology	Cat#9662; RRID: AB_331439
Rabbit polyclonal anti-cleaved Caspase-3 (Asp175) antibody (1:1,000)	Cell Signaling Technology	Cat#9661 ; RRID: AB_2341188
Mouse monoclonal anti-caspase-9 (C9) antibody (1:1,000)	Cell Signaling Technology	Cat# 9508; RRID:AB_2068620:
Mouse monoclonal anti-PARP antibody (1:1000)	BD Biosciences	Cat# 556362; RRID:AB_396387

Mouse monoclonal ATP5A1 Antibody (7H10BD4F9)	Thermo Fisher Scientific	Cat# 459240;<br>RRID:AB_2532234
Rabbit polyclonal COX IV Antibody	Cell Signaling Technology	Cat# 4844;<br>RRID: AB_2085427
Rabbit monoclonal anti-ERRa (E1G1J) antibody (1:1,000)	Cell Signaling Technology	Cat# 13826;
Mouse total OXPHOS blue native WB antibody	Abcam	Cat# ab110412;<br>RRID:AB_2847807
Rabbit monoclonal anti- β-Actin (13E5) (HRP Conjugate) antibody (1:1,000)	Cell Signaling Technology	Cat# 5125;
Goat polyclonal anti-Actin (I-19) antibody (1:5,000)	Santa Cruz Biotechnology	Cat# sc-1616;<br>RRID:AB_630836:
Donkey anti-goat IgG-HRP antibody (1:5,000)	Santa Cruz Biotechnology	Cat# sc-2020;<br>RRID:AB_631728_
Horse anti-mouse IgG-HRP antibody (1:5,000)	Cell Signaling Technology	Cat# 7076;<br>RRID:AB_330924
Goat anti-rabbit IgG-HRP antibody (1:5,000)	Cell Signaling Technology	Cat# 7074<br>RRID:AB_2099233
PE Rat Anti-mouse CD45	BD Biosciences	Cat# 553081;<br>RRID: AB_394611
APC Mouse Anti-Human CD45	BD Biosciences	Cat# 555485<br>RRID:AB_398600
**Chemicals**		
Propidium Iodide	BD biosciences	Cat# 556463
Annexin V FITC Apoptosis Detection Kit I	BD biosciences	Cat# 556547
APC Annexin V	BD biosciences	Cat# 561012
FITC BrdU Flow Kit	BD biosciences	Cat# 559619
Z-VAD-FMK	MedChemExpress	Cat# HY-16658B
Puromycin	Gibco	Cat# A1113802
Polybrene	Santa Cruz Biotechnology	Cat# NC9840454
XCT-970	Sigma Aldrich	Cat# X4753
Lipofectamine™ 3000 Transfection Reagent	Thermo Fisher Scientific	Cat# L3000-008
**Assays, primers and probes for qPCR**		
COX5A:<br>Fwd 5'-GATGCTCGCTGGGTAACATA-3'<br>Rev 5'-GGGCTCTGGAACCATATCAT-3'		NM_004255.4
COX5B<br>Fwd 5'-AAAGAAGGGACTGGACCCATA-3'<br>Rev 5'-CAGACGACGCTGGTATTGTC-3'		NM_001862.3
NDUFS3<br>Fwd 5'-CCTGTTGTCTCTGCGCTTCAA-3'<br>Rev 5'-GAAGACTCCAAACATGTCCCAG-3'		NM_004551.3
UQCRFS1<br>Fwd 5'-CCTGTGTTGGACCTGAAGC-3'<br>Rev 5'-ATAACAAACAGAAGCAGGGACAT-3'		NM_006003.3
MIC13<br>Fwd 5'-TACCAGTTCAGCCAGTACGTGTG-3'<br>Rev 5'-CTGCATTCCAGGAGTCACGGAT-3'		NM_001308240.2
MIC 10<br>Fwd 5'-TTCAGAAAGTCCCGGCTTGT-3'<br>Rev 5'-CACAAGCCCACAAGGGAACA-3'		NM_001204082.2
TMEM11<br>Fwd 5'-CCACTACATTTCCCTGCCCG-3'<br>Rev 5'-GCGTCGTACTCCACTTGGTA-3'		NM_003876.3
MIC10<br>Fwd 5'-TATGTCAAAGAGCAGGAGCAGT-3'<br>Rev 5'-GCATAACCAGGTGCTGGAGT-3'		NM_001204082.2<br>NM_001032363.4
GAPDH<br>Fwd 5'-CAAGATCATCAGCAATGCCTCC-3'<br>Rev 5'-GGTCATGAGTCCTTCCACGA-3'		NM_001357943.2
Software		
FlowJo v10.7.1	BD biosciences	RRID:SCR_008520
Prism 8	GraphPad	RRID:SCR_002798

Adobe illustrator 2021	Adobe	RRID:SCR_010279
IBM SPSS statistics 26	IBM	RRID:SCR_016479
ImageJ 1.43u	https://imagej.net/	RRID:SCR_003070
R v. 4.1.1	The R foundation	RRID:SCR_001905
Others		
Seahorse XF Base Medium	Seahorse Bioscience	Cat# 103334
Oligomycin A	Sigma Aldrich	Cat# O4876
Carbonyl cyanide 3-chlorophenylhydrazone	Sigma Aldrich	Cat# C2759
Rotenone	Sigma Aldrich	Cat# R8875
RPMI-1640	Lonza	Cat# 12-702F
DMDM	Lonza	Cat# 12-604F
Pen/Strep amphotericin B	Lonza	Cat# 17-745E
Fetal bovine serum	Gibco	Cat# 16000-044
Cell Reservoir One (with DMSO)	Nacalai tesque	Cat# 07485-44
HBSS	Welgene	Cat# LB003
Reverse Transcriptase Premix (oligo d(T) $_{15}$)	ELPis	Cat# EBT-1515C
Quantinova® SYBR® Green PCR kit (2500)	Qiagen	Cat# 208056
Lymphoprep	Stemcell	Cat# 07851
1X RBC lysis buffer	Invitrogen	Cat# 501129757
